# Bifurcation analysis of the predator–prey model with the Allee effect in the predator

**DOI:** 10.1007/s00285-021-01707-x

**Published:** 2021-12-30

**Authors:** Deeptajyoti Sen, Saktipada Ghorai, Malay Banerjee, Andrew Morozov

**Affiliations:** 1 IISER Mohali, Sahibzada Ajit Singh Nagar, India; 2 IIT Kanpur, Kanpur, India; 3 University of Leicester, Leicester, UK; 4 Severtsov Institute of Ecology and Evolution, Moscow, Russia

**Keywords:** Allee effect in predator, Partially specified models, Stability, Bifurcation, Extinction, 34C25, 92D25, 92D40

## Abstract

The use of predator–prey models in theoretical ecology has a long history, and the model equations have largely evolved since the original Lotka–Volterra system towards more realistic descriptions of the processes of predation, reproduction and mortality. One important aspect is the recognition of the fact that the growth of a population can be subject to an Allee effect, where the per capita growth rate increases with the population density. Including an Allee effect has been shown to fundamentally change predator–prey dynamics and strongly impact species persistence, but previous studies mostly focused on scenarios of an Allee effect in the prey population. Here we explore a predator–prey model with an ecologically important case of the Allee effect in the predator population where it occurs in the numerical response of predator without affecting its functional response. Biologically, this can result from various scenarios such as a lack of mating partners, sperm limitation and cooperative breeding mechanisms, among others. Unlike previous studies, we consider here a generic mathematical formulation of the Allee effect without specifying a concrete parameterisation of the functional form, and analyse the possible local bifurcations in the system. Further, we explore the global bifurcation structure of the model and its possible dynamical regimes for three different concrete parameterisations of the Allee effect. The model possesses a complex bifurcation structure: there can be multiple coexistence states including two stable limit cycles. Inclusion of the Allee effect in the predator generally has a destabilising effect on the coexistence equilibrium. We also show that regardless of the parametrisation of the Allee effect, enrichment of the environment will eventually result in extinction of the predator population.

**Supplementary Information:**

The online version supplementary material available at 10.1007/s00285-021-01707-x.

## Introduction

Modelling predator–prey interactions has always been a mainstream area in mathematical biology and theoretical ecology. Our models have evolved tremendously since the famous Lotka–Volterra system, with one realistic modification being the introduction of non-monotonous per capita growth rates to the interacting species, as opposed to the monotonically decreasing per capita growth rate seen in the logistic equation. For instance, it is currently well recognised that the growth of natural populations can be subjected to the so-called Allee effect, where the per capita growth rate increases at low species densities (Courchamp et al. 2008; Fowler and Ruxton 2002). The Allee effect can emerge at the population level due to a variety of mechanisms including enhancement in foraging efficiency, reproductive facilitation, collective defense and the modification of environmental conditions by organisms (Berec et al. 2007; Courchamp et al. 2008; Fowler and Ruxton 2002). There exist two types of Allee effect: weak and strong Allee effects. The weak Allee effect describes situations in which the per capita growth rate is increasing at small densities, but which nonetheless remains positive for low, nonzero population densities, while a strong Allee effect is characterized by a negative population growth at low densities since reproduction cannot compensate mortality rate. It has been demonstrated that including the Allee effect in predator–prey models has a strong impact on dynamics, in particular promoting population collapse and a further species extinction (Boukal et al. 2007; Hilker 2010; Lewis and Kareiva 1993; Morozov et al. 2006; Sen et al. 2012). In previous theoretical works, however, the main focus has been the scenario where there is an Allee effect in the growth rate of the prey rather than that of the predator. The scenarios where the predator growth is subject to the Allee effect are explored in the literature only partially. The aim of this paper is to contribute to bridging the gap.

The existing literature on the Allee effect in predators is scarce, and mainly focused on foraging facilitation among predators which occurs as a result of cooperative hunting (Alves and Hilker 2017; Berec 2010; Cosner et al. 1999; Sen et al. 2019). Mathematically, this implies that the functional response of the predator is an increasing function of the predator density. In particular, it was shown that it might be detrimental for cooperative hunters to be too efficient in catching prey since this may cause resource over-exploitation and eventual extinction of the predator (Alves and Hilker 2017; Sen et al. 2019). On the other hand, the Allee effect can occur in predators due to other mechanisms such as low fertilization efficiency, a lack of mating partners, sperm limitation and cooperative breeding mechanisms (Berec et al. 2007; Courchamp et al. 2008; Dennis 1989). From the modelling point of view, including an Allee effect in this case should affect the numerical response of the predator, since the food conversion efficiency becomes an increasing function of predator density, while the functional response remains unchanged. As such, the model properties and ecological predictions will be different compared to the case of the foraging facilitation scenario. Some studies have considered the Allee effect in predators due to non-foraging mechanisms, but none of them have been studied exhaustively in terms of the bifurcation structure, possible dynamical regimes and the role of parameterisations of the Allee effect in the model equations (Costa and dos Anjos 2018; Zhou et al. 2005). The latter problem may be a general issue in ecological modelling and is related to so-called structural sensitivity, which is briefly described below.

In many ecological models, predator–prey systems in particular, there is often an uncertainty regarding which precise mathematical formulation of the model functions we need to implement in the model equations (Adamson and Morozov 2013). It is often impossible to determine which particular function we need to use in the model equations to describe predation, growth, mortality, competition, etc. Several parameterisations can fit available empirical data well, and different mathematical formulations can have a valid biological rationale (Flora et al. 2011). Furthermore, implementation of close mathematical functions (both in terms of functional forms and their derivatives) in the same predator–prey model may result in different outcomes, in particular in topologically distinct bifurcation structures yielding different dynamical regimes (Adamson and Morozov 2013, 2014a). This property is called the structural sensitivity of biological models (Adamson and Morozov 2013, 2014a, b; Seo and Wolkowicz 2018). Structural sensitivity may cause major problems in terms of generality of results obtained using specific concrete formulations of model functions such as growth rates or functional responses (Adamson and Morozov 2014a; Aldebert et al. 2019). A possible way to address structural sensitivity is to allow for an unspecified formulation of some functions in the model equations with other functions being fixed, an approach is known as partially specified modelling (Wood and Thomas 1999). Implementation of the partially specified models approach is especially relevant for systems with the Allee effect in predators since this phenomenon is often caused by a variety of mechanisms, and is thus hard to describe by a single universal functional relation (Courchamp et al. 2008). Moreover, the Allee effect can depend on the spatial scale of modelling, in which case the use of a single specific mathematical formulation for the dependence of the numerical response on the overall predator density is highly questionable (Courchamp et al. 2008).

In this paper we explore a predator–prey model with an Allee effect in the predator which affects the numerical response of the predator without affecting its functional response. We consider a partially specified model, where the mathematical formulation of a strong Allee effect has only a few generic constraints to its shape. We explore the bifurcation structure of the model including saddle-node, Hopf, generalised Hopf and Bogdanov–Takens bifurcations of co-dimensions two and three. Then we construct and compare full bifurcation portraits obtained for three possible parameterisations of the Allee effect: the hyperbolic (Monod), exponential (Ivlev) and trigonometric formulations. We demonstrate that the model may exhibit structural sensitivity with respect to parameterisation of the Allee effect function. We find that adding the Allee effect results in emergence of multiple non-trival attractors in the system which can potentially explain some empirically observed alternative states in ecosystems. We argue that the Allee effect in the predator growth has a large destabilising effect on population dynamics, which has been somehow neglected previously.

## Model formulation and biological rationale

We consider a Gause type prey-predator ODE model with a specialist predator (Bazykin 1998; Hsu et al. 2001; Kuang and Freedman 1988; Turchin 2003). The model equations read as follows

1a $$\begin{aligned} \begin{aligned} \frac{dN}{dT}&=Nf(N)-g(N)P, \end{aligned} \end{aligned}$$

1b $$\begin{aligned} \begin{aligned} \frac{dP}{dT}&=e\psi (P)g(N)P - \mu P, \end{aligned} \end{aligned}$$

where *N* and *P* are the population densities of prey and predator, respectively, at time *T*. Note that in the above model, *N* and *P* can be also interpreted as the density of a resource and its consumer, respectively. In other words, the ‘predator’ in the current model can be a herbivore consuming an autotroph.

The function *f*(*N*) is the per capita growth rate of the prey which we consider here to be logistic, i.e., $f(N)= r(1-\frac{N}{K})$ and $\mu$ is the intrinsic death rate of the predator which is assumed to be constant. Functional response of predator (the rate of food consumption per predator) which we consider here to be of Holling type II and we use the following parametrisation of *g*(*N*) known as the Holling disk equation (Jost et al. 1999)

$$\begin{aligned} g(N)\,=\,\frac{aN}{1+aqN}. \end{aligned}$$

In this model, we incorporate the Allee effect in the numerical response of the predator by assuming that its food conversion efficiency $e\psi (P)$ is a function of predator density. This is different from previous models where the Allee effect was also included in the functional response of the predator (Alves and Hilker 2017; Cosner et al. 1999; Sen et al. 2019). The maximum food conversion coefficient is given by $e\, (0< e<1)$ and this value is reached at high *P*. For simplicity, we neglect direct competitive effects and interference within the predator population. We assume that the reduction of the overall growth rate at high predator densities occurs solely due to over-exploitation of food, i.e., due to a decrease in *N*. At low predator density, the per capita reproduction rate becomes smaller, which is described by the function $\psi (P)$.

Biologically, inclusion of the Allee effect in the numerical response of the predator only (and not in its functional response) can mimic a multitude of scenarios/mechanisms. A major scenario is that at a low population size it is hard to find a suitable and receptive mate and this largely reduces the reproduction rate. Mate-finding Allee effects have been found in a large number of species ranging from small insects to large birds in terrestrial ecosystems and from zooplankton to whales in the sea (Courchamp et al. 2008). Due to space limitation, here we can only list a small number of empirical examples from the literature. Namely, Mate-finding Allee effects were found in populations of flour beetles (Allee et al. 1949), muskrats (Errington 1940), whales (Ton 1948), box turtles (Mosimann 1958), condors (Mertz 1971), various zooplankton copepods (Gerritsen 1980; Kiørboe 2006), piping plovers (Strauss 1991), primates (Dobson and Lees 1989), various parasitoids (Hopper and Roush 1993), whooping cranes (Wells et al. 1998), pelagic fish (Liermann and Hilborn 2001), and elk (Larkin et al. 2002).

Another related mechanism impeding reproduction at low numbers is sperm limitation, where fertilisation of eggs requires a sufficient amount of sperm. The biological rationale is that a female needs to find a male of an optimal size, or she needs to have a sufficiently large number of males in the surroundings. The corresponding empirical examples include the blue crab (Hines et al. 2003) and the Caribbean spiny lobster (MacDiarmid and Butler 1999), where sperm limitation occurs due to excessive fishery which targets large-size males and selectively removes them from the population. The Allee effect is also possible due to low fertilization efficiency, which is observed in sessile (e.g. corals) or semi-sessile organisms (e.g. echinoderms, polychaete worms). For such broadcast spawners, at low population density, the probability of meeting of sperm and egg in water column becomes largely reduced (Aronson and Precht 2001; Courchamp et al. 2008). Our model would also mimic the Allee effect due to reproductive facilitation mechanisms. According to this scenario, only the presence of a sufficiently large number of conspecifics in the neighborhood can trigger reproduction instincts of individuals. This is the case, for example, of the flour beetle (Allee et al. 1949), queen conch (Stoner and Ray 1993), snails, and lizards (Crews and Fitzgerald 1980; Vernon 1995). Finally, our model can mimic the Allee effect due to cooperative breeding mechanisms, where at low numbers, breeding groups are less efficient in reproduction and raising young animals. Cooperative breeding is well-known in a large number of bird species (Koenig and Dickinson 2004).

Mathematically, the function $\psi (P)$ modelling the Allee effect in th predator is considered to possess the following properties:

1. $\psi (0) = 0$, since at very low densities the population cannot reproduce due to lack of mating opportunities;
2. $0 \le \psi (P) \le 1$ since $\psi (P)$ represents the proportion of the maximal possible conversion rate *e*;
3. $\psi (P)$ is an increasing function of *P*, so $\psi ^{'}(P) > 0$ for all $P \ge 0$, thus we do not include effects of intraspecific competition;
4. $\psi ^{''}(P) < 0$ for all $P \ge 0$ which signifies that the increase in the reproductive ability (population fitness), while the population size *P* is being increased, is monotonically decelerating;
5. $\psi (P) \rightarrow 1$ for large *P* (we assume the highest efficiency of reproduction at large densities).

Next we reduce the number of parameters in the model by non-dimensionalisation and introduce the following non-dimensional variables $x\,=\,\frac{N}{K}$, $y\,=\,\frac{P}{krq}$ and $t\,=\,rT$, we can transform the equations (1) to

2a $$\begin{aligned} \begin{aligned} \frac{dx}{dt}&=x\left( 1-x\right) -\frac{xy}{\beta + x}\,\equiv \,F_{1}(x,y), \end{aligned} \end{aligned}$$

2b $$\begin{aligned} \begin{aligned} \frac{dy}{dt}&=\frac{\alpha xy}{\beta + x}h(y) - my\,\equiv \,F_{2}(x,y), \end{aligned} \end{aligned}$$

with the following positive dimensionless parameters $\alpha \,=\,\frac{e}{qr}$, $\beta \,=\,\frac{1}{aqK}$ and $m\,=\,\frac{\mu }{r}$.

In the above model, the function $\psi (P)$ is transformed into a dimensionless function *h*(*y*) with the same constraints as are imposed on $\psi (P)$. As we mentioned in the Introduction, we will explore the basic properties of the model for an arbitrary mathematical formulation of *h*(*y*) (model equilibria, stability, possible generic bifurcation, etc), i.e., considering the above system as a partially specified model. We will also consider some concrete parameterisations of *h*(*y*) such as $h(y)=\frac{y}{\delta + y}$ (Monod parametrisation), $h(y)=1-e^{-\frac{y}{\delta }}$ (Ivlev parametrisation) and $h(y)=\tanh (\frac{y}{\delta })$ (hyperbolic tangent parameterisation) to construct a full bifurcation portrait and explore the sensitivity of the model dynamics to mathematical formulation of the Allee effect. Finally, we verify how sensitive the model is with respect to small perturbations of *h*(*y*) which still preserve assumptions (A1)–(A5).

## Model equilibria and their stability

### Possible equilibria in the system

We start our investigation by exploring the number and the location of system equilibria for an arbitrary formulation of the Allee effect *h*(*y*). It is easy to see that model (2) always has one trivial equilibrium point $E_{0}\,=\,(0,0)$ and one axial (predator-free) equilibrium point $E_{1}\,=\,(1,0)$.

An interior equilibrium point $E^{*}\,=\,(x^{*},y^{*})$ will be a point of intersection of the following two non-trivial nullclines in the interior of first quadrant

3a $$\begin{aligned} \begin{aligned} f^{1}(x,y)&\equiv \, 1-x-\frac{y}{x+\beta }\,=\,0, \end{aligned} \end{aligned}$$

3b $$\begin{aligned} \begin{aligned} f^{2}(x,y)&\equiv \, \frac{\alpha x}{x+\beta }h(y)-m\,=\,0. \end{aligned} \end{aligned}$$

For the feasibility of $y^{*}$, we must have $0< x^{*} <1$ (see Eq. (3a)) and from this condition we can verify that $0<y^*\le \frac{(1+\beta )^2}{4}$. We solve (3b) for *x* to obtain the equation for the non-trivial predator nullcline

4 $$\begin{aligned} x= & {} \frac{m \beta }{\alpha h(y)-m}. \end{aligned}$$

For the feasibility of $x^{*}$, we must have $y^{*} > h^{-1}(\frac{m}{\alpha })$. Since the Allee effect function *h*(*y*) is bounded by 1, from $h(y)=\frac{m(x+\beta )}{\alpha x}$ we find $x^{*} > \frac{m \beta }{\alpha -m}$ with $\alpha >m$. We differentiate Eq. (4) with respect to *y* to obtain

$$\begin{aligned} \frac{dx}{dy}\,=\,-\frac{\alpha m \beta h^{'}(y)}{(\alpha h(y)-m)^2} <0 \quad as \quad h^{'}(y) > 0. \end{aligned}$$

**Fig. 1:**
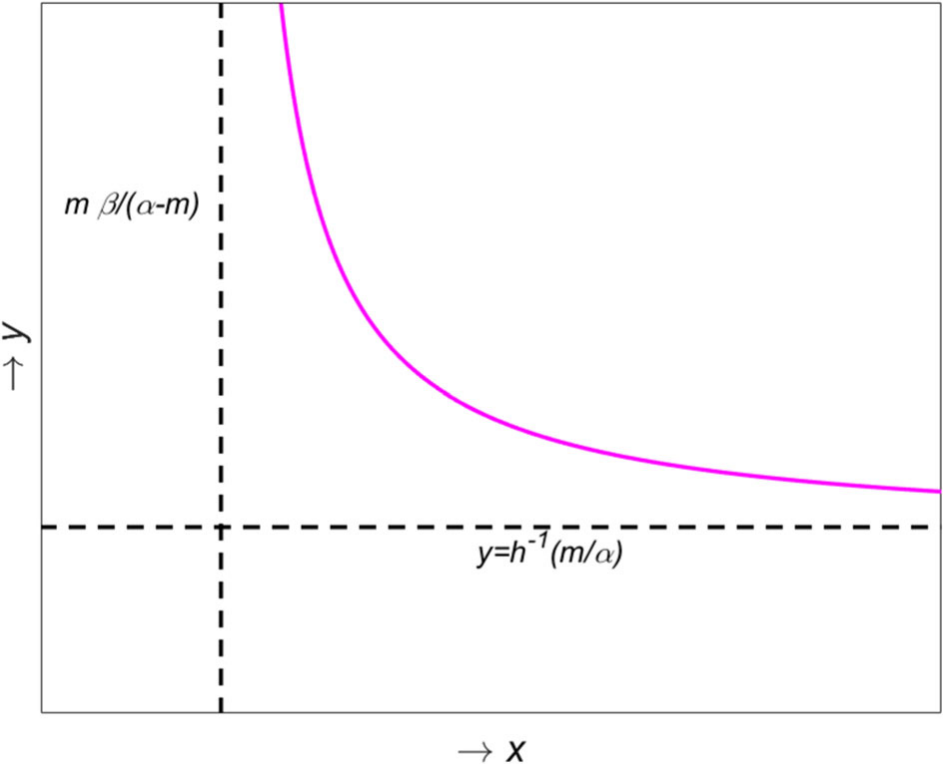
Qualitative behviour of the predator nullcline in model (2)

For the second derivative of the predator nullcline we have

$$\begin{aligned} \frac{d^{2}x}{dy^{2}}\,=\,\frac{\alpha \beta m}{(\alpha h(y)-m)^2}\left[ \frac{2 \alpha (h^{'}(y))^2}{\alpha h(y)-m}-h^{''}(y)\right] > 0, \end{aligned}$$

as $h^{''}(y) < 0$ and $\alpha h(y) > m$. From the above we derive that the predator nullcline (3b) is strictly decreasing as a function of *x* and it is always convex. Furthermore, the curve lies in the region where $x > \frac{m \beta }{\alpha -m}$ and $y > h^{-1}(\frac{m}{\alpha })$. Taking into account the above properties, a possible shape of the predator nullcline is shown in Fig. 1; the dashed lines represents the vertical and the horizontal asymptotes.

**Fig. 2:**
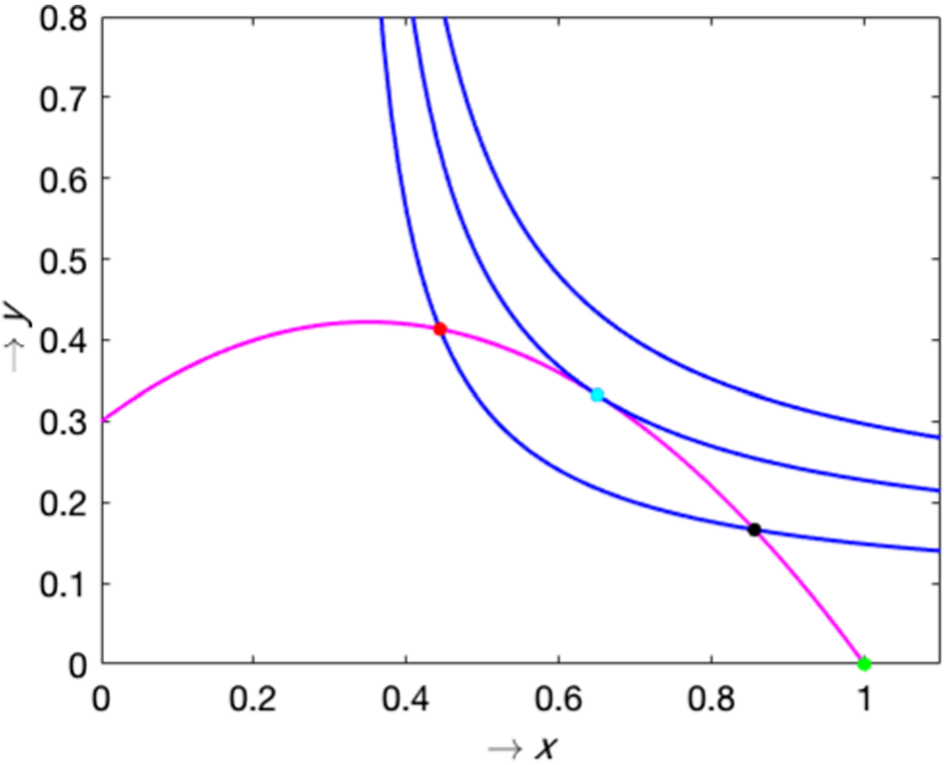
Relative position of nullclines in model (2) constructed for the Allee effect parametrisation given by the Monod function $h(y)=\frac{y}{\delta + y}$ with $\delta \,=\,0.4$ (no equilibrium), $\delta \,=\,0.315$ (a single equibrium), and $\delta \,=\,0.1$ (two equilibria points): one is a saddle point (black dot), the other is a topological focus (red dot). Other parameters are $\alpha \,=\,1.8$, $\beta \,=\,0.4$ and $m\,=\,0.5$

From the geometric properties of the nullclines one can see that there will be at most two points of intersection (between the non-trivial nullclines in the interior of the first quadrant) and so there can be at most two interior equilibria. An example of intersection of the model nullclines for the parameterisation of *h*(*y*) given by $h(y)=\frac{y}{\delta + y}$ is shown in Fig. 2. One can see that a gradual increase in $\delta$ (which defines characteristic predator densities at which the Allee effect has a pronounced strength) from small to large values results a saddle-node bifurcation which is described in detail in the next sections. Note that this property is observed for the other two parametrisations of *h*(*y*) considered. Clearly, in the absence of the Allee effect, only one non-trivial equilibrium is possible, corresponding to the intersection of the vertical line $x=m\beta /(\alpha -m)$ and the prey nullcline (3a).

### Stability of equilibria

Here we explore the stability for the equilibria of model (2). The following proposition defines the stability of the axial equilibria.

#### Proposition 1

For any choice of *h*(*y*) satisfying assumptions (A1)-(A5)

(i) The trivial equilibrium point $E_{0}$ is a saddle;
(ii) The axial equilibrium point $E_{1}$ is locally asymptotically stable.

#### Proof

The Jacobian matrix of model (2) at any point is given by

5 $$\begin{aligned} J(x,y)= & {} \begin{bmatrix} 1-2x-\frac{y}{\beta + x}+\frac{xy}{(\beta + x)^2} &{} -\frac{x}{\beta + x}\\ \frac{\alpha \beta y h(y)}{(\beta + x)^2} &{} \frac{\alpha x}{\beta + x}h(y) + \frac{\alpha x y}{\beta + x}h^{'}(y)-m \end{bmatrix}. \end{aligned}$$

(i) The eigenvalues of the Jacobian matrix at $E_{0}$ are 1 and $-m$. Therefore it is a saddle point irrespective of the choice of *h*(*y*), having a stable manifold along *y*-axis and an unstable manifold along *x*-axis.
(ii) The axial equilibrium point $E_{1}$ is locally asymptotically stable (a stable node) as the eigenvalues of the Jacobian matrix are $-1$ and $-m$ for any choice of *h*(*y*).

$$\square $$

An important conclusion is that, in the presence of an Allee effect in the predator, achieving a very low population densities by the predator will result in its eventual extinction, so the Allee effect is strong.

Next we explore the stability of the interior equilibria. As follows from the previous section, model (2) admits at most two interior equilibrium points which we denote by $E_{1*}(x_{1*},y_{1*})$ and $E_{2*}(x_{2*},y_{2*})$ such that $0\,<\,x_{1*}\,<\,x_{2*}\,<\,1$. The Jacobian matrix evaluated at $E_{*}\,=\,(x_{*},y_{*})$ can be expressed as

6 $$\begin{aligned} J(E^{*})&= \begin{bmatrix} xf^{1}_{x} &{} xf^{1}_{y}\\ yf^{2}_{x} &{} yf^{2}_{y} \end{bmatrix}_{(x_{*},y_{*})}, \end{aligned}$$

where we have used $f^{1}(x_{*},y_{*})\,=\,0$ and $f^{2}(x_{*},y_{*})\,=\,0$. Since $f^{1}$ and $f^{2}$ are smooth functions, we can differentiate both expressions (3) to obtain

$$\begin{aligned} f^{1}_{x}= & {} -f^{1}_{y}\frac{dy^{(f^{1})}}{dx}, f^{2}_{x} = -f^{2}_{y}\frac{dy^{(f^{2})}}{dx}, \end{aligned}$$

where $\frac{dy^{(f^{1})}}{dx}$ and $\frac{dy^{(f^{2})}}{dx}$ are the tangent lines to the nullclines $f^{1}(x,y)\,=\,0$ and $f^{2}(x,y)\,=\,0$, respectively. We substitute the above expressions into the Jacobian matrix

7 $$\begin{aligned} J(E^{*})&= \begin{bmatrix} -xf^{1}_{y} \frac{dy^{(f^{1})}}{dx} &{}\quad xf^{1}_{y}\\ -yf^{2}_{y} \frac{dy^{(f^{2})}}{dx} &{}\quad yf^{2}_{y} \end{bmatrix}_{(x_{*},y_{*})}. \end{aligned}$$

Therefore, for the determinant of the Jacobian we obtain

8 $$\begin{aligned} Det (J(E^{*}_{i}))&= \left[ xyf^{1}_{y}f^{2}_{y}\left( \frac{dy^{(f^{2})}}{dx}-\frac{dy^{(f^{1})}}{dx}\right) \right] _{(x_{*},y_{*})}. \end{aligned}$$

Now $f^{1}_{y}(x,y)\,=\,-\frac{1}{\beta + x} < 0$ and $f^{2}_{y}(x,y)\,=\,\frac{\alpha x}{\beta + x}h^{'}(y) > 0$ as $h^{'}(y) > 0$. Substituting the above derivatives we have

9a $$\begin{aligned} \begin{aligned} \frac{dy^{(f^{1})}}{dx}&= 1-\beta -2x \,=\,2(x_{m}-x),\end{aligned} \end{aligned}$$

9b $$\begin{aligned} \begin{aligned} \frac{dy^{(f^{2})}}{dx}&= -\frac{\beta h(y)}{\beta + x}\frac{1}{xh^{'}(y)} < 0 , \end{aligned} \end{aligned}$$

where $x_{m}\,=\,\frac{1-\beta }{2}$ is the *x*-coordinate of the point where the nullcline $f^{1}(x,y)\,=\,0$ attains its maximum within the first quadrant (for $0<\beta <1$).

Depending upon the positions of two points of intersections between the two nontrivial nullclines (cf. (3)) with respect to the point of maximum on the prey nullcline, we can consider following two cases,

**Case:1**  $0< x_{m}< x_{1*}< x_{2*} < 1$ and **Case:2**  $0< x_{1*}< x_{m}< x_{2*} < 1$.

**Fig. 3:**
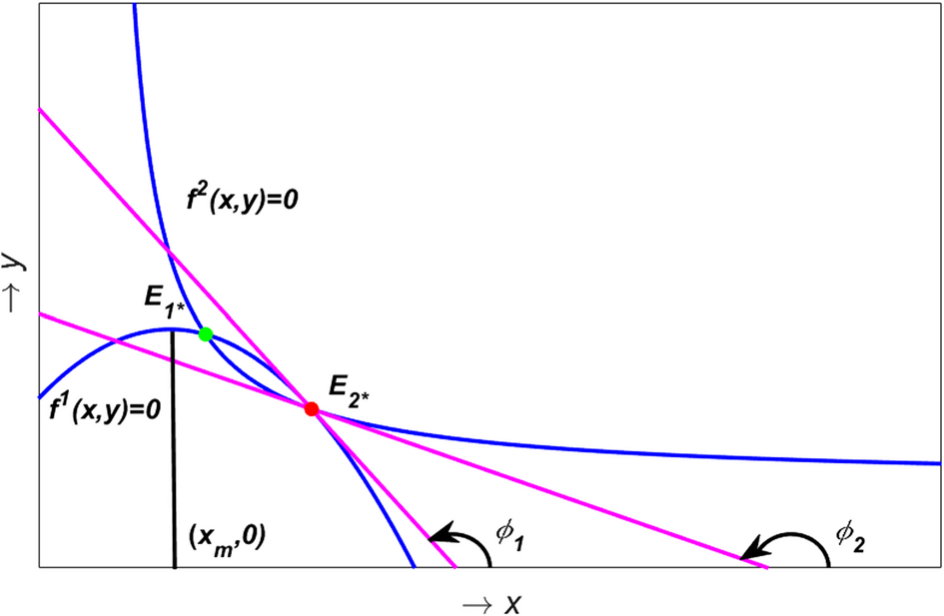
Determining the type and stability of interior equilibria in model (2). For detail see the text

**Case:1**  Suppose $\phi _1$ and $\phi _2$ are angles made by the tangents to $f^1(x,y)\,=\,0$ and $f^2(x,y)\,=\,0$ at $E_{2*}$. Then from Fig. 3 one can see that $\frac{\pi }{2}\,<\,\phi _1\,<\,\phi _2\,<\,\pi$ which implies

10 $$\begin{aligned} \left. \frac{dy^{(f^2)}}{dx}\right| _{E_{2*}}\,>\,\left. \frac{dy^{(f^1)}}{dx}\right| _{E_{2*}}. \end{aligned}$$

Therefore from the above inequality and expression (8) we get $Det (J(E_{2*})) < 0$. Hence $E_{2*}$ is a saddle point.

We can proceed in a similar fashion and use the fact that (this is not shown in Fig.  3 for brevity)

11 $$\begin{aligned} \left. \frac{dy^{(f^1)}}{dx}\right| _{E_{1*}}\,>\,\left. \frac{dy^{(f^2)}}{dx}\right| _{E_{1*}} \end{aligned}$$

and to prove that $\text {Det}\left( J(E_{1*})\right) \,>\,0$, i.e., $E_{1*}$ is not a saddle point. The stability of $E_{1*}$ is determined by the sign of $f_x^1(x_{1*},y_{1*})+f_y^2(x_{1*},y_{1*})$. Note that

12a $$\begin{aligned} f_x^1(x_{1*},y_{1*})= & {} 2(x_{m}-x_{1*})< 0 \quad as \quad x_{m} < x_{1*}, \end{aligned}$$

12b $$\begin{aligned} f_y^2(x_{1*},y_{1*})= & {} \frac{\alpha x_{1*}h'(y_{1*})}{\beta + x_{1*}}\,>\,0. \end{aligned}$$

Hence $E_{1*}$ is locally asymptotically stable if

13 $$\begin{aligned} f_x^1(x_{1*},y_{1*})+f_y^2(x_{1*},y_{1*})\,<\,0. \end{aligned}$$

**Case:2** In this case we can also prove that $E_{2*}$ is a saddle point proceeding in a similar manner as above. For the stability of $E_{1*}$ we have from (9a), $\left. \frac{dy^{(f^1)}}{dx}\right| _{E_{1*}} > 0$. Hence we get from (8), $Det (J(E_{1*})) > 0$. Also

14a $$\begin{aligned} f_x^1(x_{1*},y_{1*})= & {} 2(x_{m}-x_{1*})> 0 \quad as \quad x_{m} > x_{1*}, \end{aligned}$$

14b $$\begin{aligned} f_y^2(x_{1*},y_{1*})= & {} \frac{\alpha x_{1*}h'(y_{1*})}{\beta + x_{1*}}\,>\,0. \end{aligned}$$

As $tr (J(E_{1*}))\,=\,f_x^1(x_{1*},y_{1*}) + f_y^2(x_{1*},y_{1*}) > 0$, hence $E_{1*}$ is unstable.

To conclude, the interior equilibrium $E_{2*}$ is always a saddle point, whereas $E_{1*}$ is a topological focus which depending on parameters can be either stable or unstable.

## Local bifurcations in the model

Here we consider possible local bifurcations in model (2).

### Saddle-node bifurcation

Suppose ${\overline{E}}({\overline{x}},\,{\overline{y}})$ is the point at which two non-trivial nullclines touch each other in the first quadrant when a bifurcation parameter of the model is being varied. The slope of the tangents to the curves at ${\overline{E}}$ become equal. This signifies that

15 $$\begin{aligned} \left. \frac{dy^{(f^1)}}{dx}\right| _{({\overline{x}},\,{\overline{y}})}\,=\, -\left. \frac{f_x^1}{f_y^1}\right| _{({\overline{x}},\,{\overline{y}})}\,=\, -\left. \frac{f_x^2}{f_y^2}\right| _{({\overline{x}},\,{\overline{y}})}\,=\, \left. \frac{dy^{(f^2)}}{dx}\right| _{({\overline{x}},\,{\overline{y}})}, \end{aligned}$$

and hence we have

16 $$\begin{aligned} \left. f_x^1f_y^2-f_y^1f_x^2\right| _{({\overline{x}},\,{\overline{y}})}\,=\,0. \end{aligned}$$

In this case $\text {Det}\left( J({\overline{E}})\right) \,=\,0$ and ${\overline{E}}$ becomes a non-hyperbolic equilibrium point. This situation corresponds to a saddle-node bifurcation in the model. We explore this bifurcation in more detail.

As an example, we consider *m* to be the bifurcation parameter and denote by $m\,\equiv \,m_{SN}$ the bifurcation point. The eigenvectors of both the matrix $J({\bar{E}})$ and its transpose corresponding to the zero eigenvalue are, respectively given by $v\,=\,[1,1-\beta -2{\bar{x}}]^{t}$ and $w\,=\,[\alpha {\bar{y}}h^{'}({\bar{y}}),1]^{t}$. We need to check the transversality conditions for a saddle-node bifurcation (Perko 2013). We denote $F\,=\,(F_{1}(x,y),F_{2}(x,y))^{t}$ and we further follow the same notation of (Perko 2013) to obtain

$$\begin{aligned} w^{t}F_{m}({\bar{E}};m=m_{SN})= & {} -1\,\ne \,0,\\ w^{t}D^{2}F({\bar{E}};m=m_{SN})(v,v)= & {} -\frac{2\alpha {\bar{x}}{\bar{y}}h^{'}({\bar{y}})}{\beta + {\bar{x}}}-\frac{\beta {\bar{y}}h({\bar{y}})}{(\beta + {\bar{x}})^2}\\&\left[ 2 + \frac{2\beta }{{\bar{x}}(\beta + {\bar{x}})} - \frac{\beta h({\bar{y}})h^{''}({\bar{y}})}{{\bar{x}}(\beta + {\bar{x}})(h^{'}({\bar{y}}))^2}\right] \,<\,0, \end{aligned}$$

as $h^{''}(y) < 0$. Hence the transversality conditions are always satisfied and variation of *m* results in a saddle-node bifurcation. Similar results can be obtained by varying other model parameters.

### Hopf bifurcation

In the previous subsection we show that the two interior equilibrium points are generated through a saddle-node bifurcation. The non-saddle interior equilibrium ($E_{1*}$) can be stable or unstable depending on model parameters. It loses its stability when the sign of the trace of the Jacobian matrix has changed through zero (from negative to positive) via a Hopf bifurcation. In this section we show that system (2) undergoes a Hopf bifurcation when a model parameter is varied. Here we choose $\beta$ as a bifurcation parameter. The Jacobian matrix at $E_{1*}$ is given by

$$\begin{aligned} J(E_{1*})= & {} \begin{bmatrix} \frac{x_{1*}}{\beta +x_{1*}}(1-\beta -2x_{1*}) &{} -\frac{x_{1*}}{\beta +x_{1*}}\\ [0.5em] \frac{\alpha \beta y_{1*}h(y_{1*})}{(\beta +x_{1*})^2} &{} \frac{\alpha x_{1*}y_{1*}}{\beta +x_{1*}}h^{'}(y_{1*}) \end{bmatrix}. \end{aligned}$$

Now let us assume that $\beta \,=\,1-2x_{1*}+\alpha y_{1*}h^{'}(y_{1*})\,\equiv \,\beta _{H}$. This is an implicit expression for $\beta$ as the components of the equilibrium point containg $\beta$ as well. Now we assume that the following three conditions are satisfied at $\beta \,=\beta _H$,

1. $$T_{H}\,=\,tr (J(E_{1*};\beta \,=\,\beta _{H}))\,=\,0$$,
2. $$\Delta _{H}\,=\,det (J(E_{1*});\beta \,=\,\beta _{H}) > 0$$,
3. If $\lambda (\beta )$ is the complex eigenvalue of $J(E_{1*})$ then $\left. \frac{d}{d\beta }\left( Re (\lambda (\beta ))\right) \right| _{\beta =\beta _H}\,\ne \,0.$

Then $E_{1*}$ loses its stability through a Hopf bifurcation at $\beta \,=\,\beta _{H}$.

Assuming $Re (\lambda (\beta ))$ is real part of a complex eigenvalue of $J(E_{1*})$, we can write,

$$\begin{aligned} Re (\lambda (\beta ))=tr(J(E_{1*}))/2\,=\,\frac{x_{1*}}{2(\beta +x_{1*})}\left\{ 1-\beta -2x_{1*}+\alpha y_{1*}h^{'}(y_{1*})\right\} . \end{aligned}$$

Now $Re (\lambda (\beta ))$ is equal to zero when $\beta \,=\,\beta _H$. Differentiating $Re (\lambda (\beta ))$ with respect to $\beta$ we find that

17 $$\begin{aligned} \frac{d}{d\beta }\{Re (\lambda (\beta ))\}= & {} \frac{x_{1*}}{2(\beta + x_{1*})}\left\{ -1-2\frac{dx_{1*}}{d\beta }+\alpha \frac{dy_{1*}}{d\beta }\left( h^{'}(y_{1*})+y_{1*}h^{''}(y_{1*})\right) \right\} \nonumber \\&+\frac{\beta }{2(\beta +x_{1*})^2}\frac{dx_{1*}}{d\beta }\left\{ 1-\beta -2x_{1*}+\alpha y_{1*}h^{'}(y_{1*})\right\} . \end{aligned}$$

Now as $E_{1*}$ satisfies (3a) we have,

$$\begin{aligned} \frac{dy_{1*}}{d\beta }\,=\,1-x_{1*}+\frac{dx_{1*}}{d\beta }\left( 1-2x_{1*}-\beta \right) . \end{aligned}$$

Finally using the fact $1-\beta -2x_{1*}+\alpha y_{1*}h^{'}(y_{1*})\,=\,0$ at $\beta \,=\,\beta _H$ and above result in (17) we get

$$\begin{aligned} \left. \frac{d}{d\beta }\{Re (\lambda (\beta ))\}\right| _{\beta \,=\,\beta _{H}}= & {} \frac{x_{1*}}{2(\beta _{H} + x_{1*})}\left[ -1+\alpha (1-x_{1*})\left( h^{'}(y_{1*})+y_{1*}h^{''}(y_{1*})\right) -\right. \\&\left. \frac{dx_{1*}}{d\beta }\left( 2-\alpha (1-2x_{1*}-\beta _{H})(h^{'}(y_{1*})+y_{1*}h^{''}(y_{1*}))\right) \right] _{\beta =\beta _H}. \end{aligned}$$

The above expression should be checked for the given mathematical formulation of the Allee effect *h*(*y*). In particular, we have numerically verified that this quantity is non-zero at the Hopf bifurcation threshold for the parameterisations considered here: the Monod, Ivlev and trigonometric functions.

### Generalized Hopf (Bautin) bifurcation

In this subsection we consider a co-dimension two bifurcation called a Bautin or generalized Hopf (GH) bifurcation. This bifurcation occurs when the interior non-saddle equilibrium has purely imaginary eigenvalues and the first Liapunov number becomes zero. We consider $\beta$ and *m* as bifurcation parameters. Therefore in $\beta$–*m* parametric plane, there is a critical point which lies on the Hopf bifurcation curve. In the next proposition we will show that the model undergoes a GH bifurcation by choosing $\beta$ and *m* as bifurcation parameters.

#### Proposition 2

Model (2) undergoes a Bautin (generalized Hopf) bifurcation around the interior equilibrium point ${\hat{E}}\,=\,({\hat{x}},{\hat{y}})$ at the bifurcation threshold $(\beta _{GH},m_{GH})$ whenever the following conditions hold

1. $$\Delta _{GH}\,=\,det (J({\hat{E}});\beta \,=\,\beta _{GH},\,m\,=\,m_{GH}) > 0$$,
2. $$T_{GH}\,=\,tr (J({\hat{E}});\beta \,=\,\beta _{GH},\,m\,=\,m_{GH})\,=\,0$$,
3. $$l({\hat{E}};\beta \,=\,\beta _{GH},\,m\,=\,m_{GH}))\,=\,0$$,

where *l* is the first Liapunov number.

#### Proof

See supplementary material SM1. $\square$

Examples of the above type of bifurcation for several parameterisations of *h*(*y*) are provided in Sect. 5.

### Bogdanov–Takens bifurcation

Another type of co-dimension two local bifurcation observed in model (2) is a Bogdanov–Takens (BT) bifurcation. In a two dimensional parametric plane, this bifurcation occurs at a point where a Hopf bifurcation curve meets a saddle-node bifurcation curve tangentially. In the previous subsection, we chose $\beta$ as the bifurcation parameter for the Hopf bifurcation and *m* for the saddle-node bifurcation. Therefore we will consider $\beta$ and *m* as bifurcation parameters for the BT bifurcation and suppose that model (2) exhibits a BT bifurcation at ${\bar{E}}\,=\,({\bar{x}},{\bar{y}})$ and the parametric thresholds are denoted by $(\beta ,m)\,=\,(\beta _{BT},m_{BT})$. From the general bifurcation theory (Perko 2013) it is known that ${\bar{E}}$ satisfies the equations of nullclines (3) and also the Jacobian matrix is similar to $$\begin{bmatrix} 0 &{} 1\\ 0 &{} 0 \end{bmatrix}$$ at ${\bar{E}}$ for the parameter threshold $(\beta \,=\,\beta _{BT},m\,=\,m_{BT})$. The following proposition provides the conditions for model (2) to undergo a Bogdanov–Takens bifurcation.

#### Proposition 3

If we choose $\beta$ and *m* as bifurcation parameters, then system (2) undergoes a Bogdanov–Takens bifurcation around the interior equilibrium point ${\bar{E}}$ whenever the following conditions hold

1. $$tr (J({\bar{E}};\beta \,=\,\beta _{BT},\,m\,=\,m_{BT}))\,=\,0$$ ,
2. $$det (J({\bar{E}};\beta \,=\,\beta _{BT},\,m\,=\,m_{BT}))\,=\,0$$.

#### Proof

See supplementary material SM 2 for detail. $\square$

We found that model (2) may undergo a Bogdanov–Takens bifurcation of either co-dimension 2 or co-dimension 3. The latter requires an extra condition of degeneracy given in the supplementary material. Note that a co-dimension 3 Bogdanov–Takens bifurcation, if it exists, should be of the type involving a double equilibrium point (Dumortier et al. 1987). Indeed, the other type of this bifurcation—known as the cusp—would require a triple equilibrium point which is impossible for this model as shown in Sect. 3. For the same reason, a co-dimension 4 Bogdanov–Takens bifurcation is impossible in this system. Examples of Bogdanov–Takens bifurcation of co-dimension 2 and 3 for particular parameterisations of *h*(*y*) are provided in the next section.

## Parametric diagrams and phase portraits

**Fig. 4:**
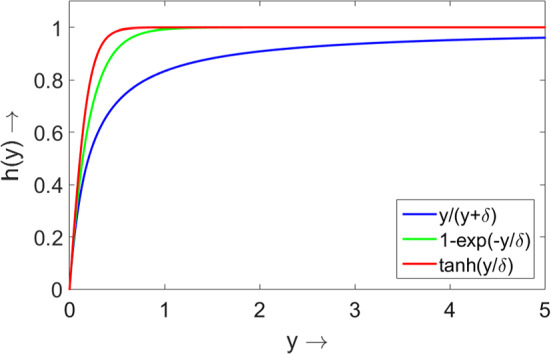
Graphs of parameterisations of the Allee effect in the predator given by the Monod ($y/(y+\delta )$), the Ivlev ($1-\exp (-y/ \delta )$) and the trigonometric ($\tanh (y/ \delta )$) functions; $\delta =0.2$

In this section, we construct global parametric diagrams for the considered model for three different mathematical formulations of the Allee effect *h*(*y*) given by the Monod, Ivlev and trigonometric tangent functions. Note that all of them satisfy assumptions (A1)–(A5). Examples of all three curves constructed for $\delta =0.2$ are shown in Fig.  4. Note that for the plotted functions the initial slopes and their asymptotic values for large *y* are the same.

The model contains 4 parameters, so it is convenient to present our results in a 3 dimensional parametric space and then explore the alteration to the portrait by varying a fourth parameter. We construct portraits in the $(\alpha ,\delta ,m)$ space with a further variation of $\beta$. For all considered formulations of *h*(*y*), the parameter $\delta$ can be interpreted as the intensity of the Allee effect. In particular, in the case where $\delta$ vanishes the system becomes the classical Rosenzweig–MacArthur predator–prey model.

**Fig. 5:**
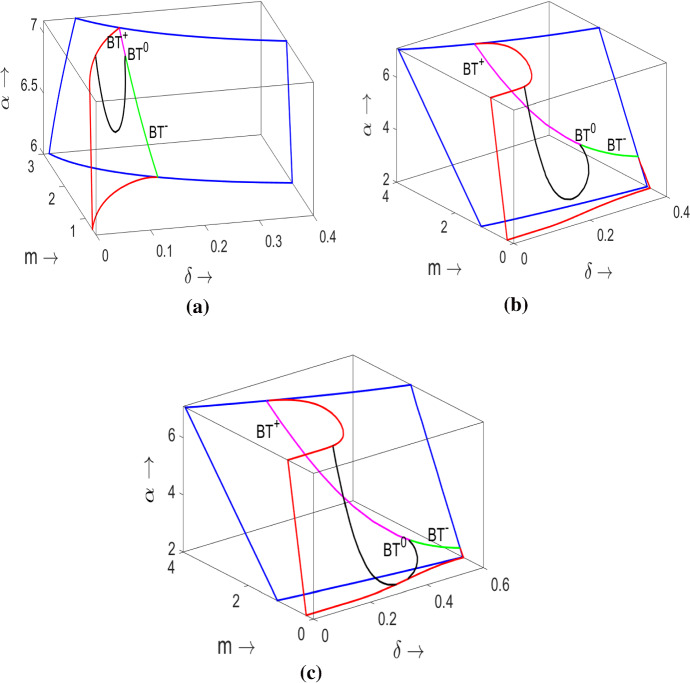
Three dimensional diagram $(\alpha ,\delta ,m)$ of model (2) for the Allee effect parameterised by: **a** the Monod response $h(y)=y/(\delta +y)$; **b** the Ivlev response $h(y)=1-e^{-\frac{y}{\delta }}$; **c** the trigonometric tangent function $h(y)=\tanh (\frac{y}{\delta })$. In each diagram, $\beta =0.8$. The explanation of the surfaces and curves is in the text

Examples of parametric portraits for the three functional forms of *h*(*y*) are given in Fig. 5, in each case $\beta =0.8$ is kept fixed. We show the skeletons of the parametric portraits given by local bifurcations: to avoid overloading the diagram, we do not include the non-local bifurcations which are shown in the corresponding cross sections in next figures. The saddle-node bifurcation surface is denoted by the blue curves. These curves show intersections of the saddle-node bifurcation surface with boundaries of the parameteric diagram. The intersection of the Hopf bifurcation surface with the boundaries is denoted by red curves. The saddle-node and Hopf surfaces intersect along the Bogdanov–Takens bifurcation curve which consists of green and magenta coloured parts: the magenta colour corresponds to a Bogdanov–Takens bifurcation of codimension 2 with a positive product of the state variables in the normal form (see SM 2 for detail) and is denoted as $BT^+$; the green part of the curve gives Bogdanov–Takens bifurcation of codimension 2 with this product having negative sign and is denoted as $BT^-$. The black curve represents the location of Generalised Hopf points on the Hopf bifurcation surface. This curve emerges from the point of Bogdanov–Takens bifurcation of codimension 3 (denoted as $BT^0$). From comparison of the diagrams in Fig. 5, we conclude that the global bifurcation structure in the parametric space remains the same topologically for all three formulations of *h*(*y*).

**Fig. 6:**
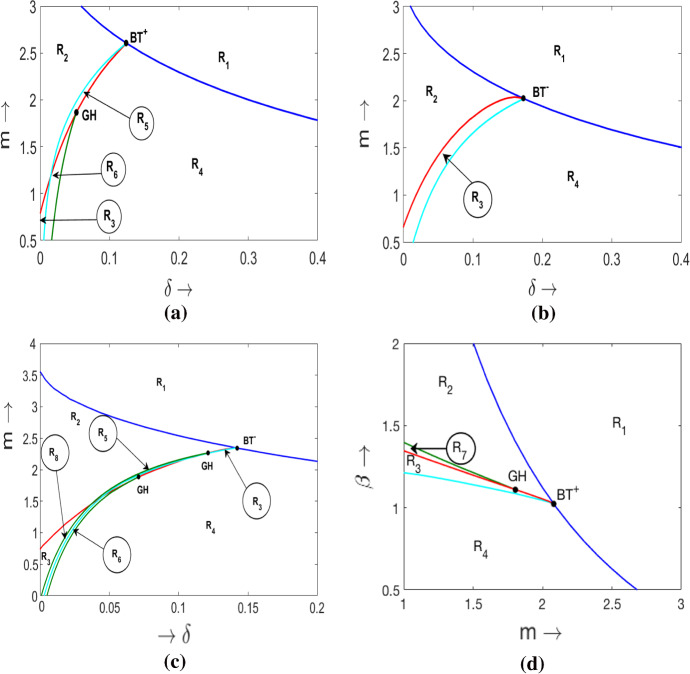
**a**–**c** Examples of bifurcation diagrams in $\delta$–*m* parametric plane for the Monod parameterisation of the Allee effect in predator plotted for $\alpha$ above and below the $BT^0$ point shown in Fig. 5a. The parameter values are: **a** $\alpha \,=\,7.1$; **b** $\alpha \,=\,6.0$ and **c** $\alpha \,=\,6.6$; in all cases $\beta \,=\,0.8$. **d** Two dimensional diagram $(\beta , m)$ constructed for $\alpha =7.1$ and $\delta =0.2$. In each diagram, the dark blue curve is a saddle-node bifurcation curve. The red curve is a Hopf bifurcation curve. The green curve is the curve of a fold bifurcation of limit cycles. The cyan curve describes a homoclinic bifurcation. The meaning of the regions $R_i$ is explained in Fig.7 and in the main text

**Fig. 7:**
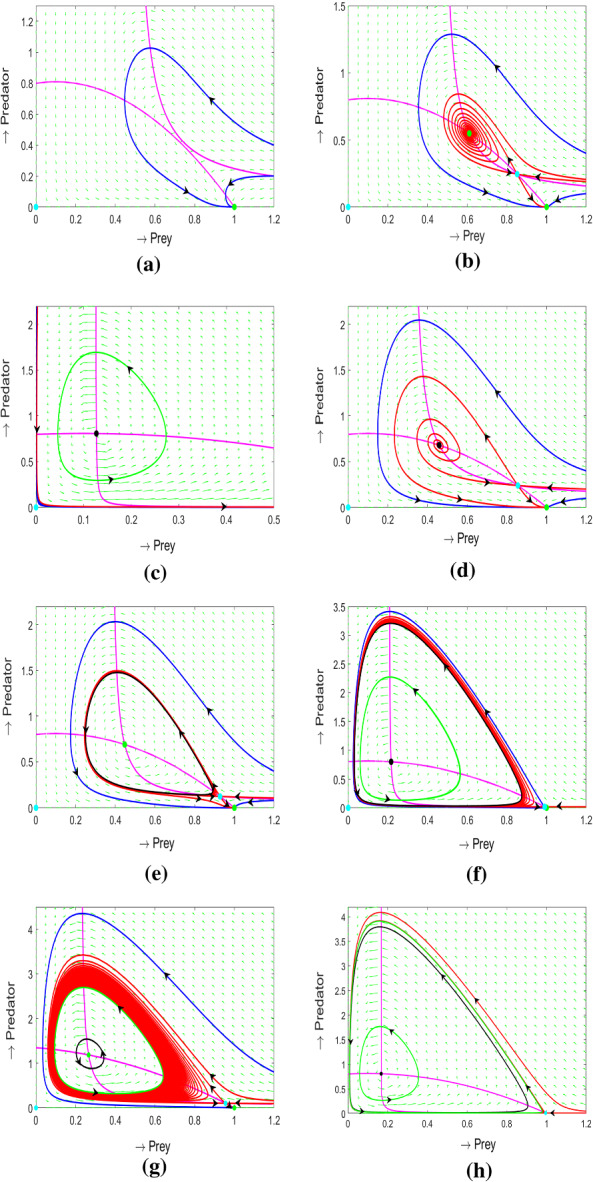
Phase portraits for the model with the Monod form of *h*(*y*). **a**–**f** are constructed for $\alpha \,=\,7.1$, $\beta \,=\,0.8$; the other parameters are **a** $\delta \,=\,0.12$, $m\,=\,2.66$, region ($R_1$); **b** $\delta \,=\,0.1$, $m\,=\,2.6$, region ($R_2$); **c** $\delta \,=\,0.01$, $m\,=\,0.96$, region ($R_3$); **d** $\delta \,=\,0.2$, $m\,=\,2$, region ($R_4$); **e** $\delta \,=\,0.085$, $m\,=\,2.67$, region ($R_5$); **f** $\delta \,=\,0.03$, $m\,=\,1.456$, region ($R_6$). **g** is plotted for $\alpha =7.1$; $\delta =0.2$; $\beta =1.347$; $m=1$, region ($R_7$); **h** is plotted for $\alpha =6.6$, $\beta =0.8$, $\delta =0.02$, $m=1.09$, region ($R_8$)

To better understand the parametric structure and feasible phase portraits in the model, we explored two-dimensional cross sectional diagrams for a constant $\alpha$ and $\beta$. In the main text, we present the diagrams for the Monod formulation of *h*(*y*). The diagrams for the other functional forms of *h*(*y*) are shown in the supplementary material (SM 3). An example of a $(\delta ,m)$ diagram constructed for $\alpha$ above the $BT^0$ point is shown in Fig.  6a; the other two $(\delta ,m)$ diagrams in the same figure are constructed for $\alpha$ below the $BT^0$ point. Figure 6b describes the situation where the $(\delta ,m)$ plane does not intersect the GH bifurcation curve, the opposite case is shown in Fig. 6c. The corresponding phase portraits of the model are given in Fig. 7.

From Fig. 6a–c one can see that for large values of *m* and $\delta$ (region $R_1$) there are no coexistence equilibria in the system: the only (global) attractor is the state $E_{1}$, where only the prey survives, whereas the predator goes to extinction. The corresponding phase portrait is shown in Fig. 7a.

Reduction in the strength of the Allee effect (small $\delta$ and high rates of mortality *m*) results in the emergence of a pair of equilibrium points: a saddle $E_{1*}$ and a node $E_{2*}$ (region $R_2$). The non-saddle point is only locally stable: its basin of attraction is limited by that of the axial equilibrium point $E_{1}$, which is shown in Fig. 7b. For large values of *m* (on the right hand side of a BT point), a decrease in $\delta$ will result in a saddle-node bifurcation where the non-saddle point $E_{1*}$ will be unstable (region $R_4$). In this case, the global attractor will be the prey only state $E_{1}$ (the phase portrait is shown in Fig. 7d). The loss of stability of $E_{1*}$ when crossing the Hopf bifurcation curve around the BT point depends on the sign of the BT point. For $BT^+$ (Fig. 6a) transition from $R_2$ to $R_4$ occurs via region $R_5$ by crossing the homoclinic loop bifurcation curve. A locally stable interior equilibrium $E_{1*}$ bcomes surrounded by an unstable cycle which forms its basin of attraction (Fig. 7e). All trajectories starting outside this cycle will be attracted to the prey only state $E_1$. The transition from region $R_5$ to region $R_2$ occurs via a homoclinic loop bifurcation. For $BT^-$ (Fig. 6b), the transition from $R_2$ to $R_4$ occurs via region $R_3$ by crossing a supercritical Hopf bifurcation curve. In region $R_3$, an unstable internal equilibrium $E_{1*}$ is surrounded by a stable limit cycle (Fig. 7c). One can see that in Fig. 6a, for smaller *m*, a decrease in $\delta$ from region $R_4$ results in a fold bifurcation of limit cycles. In region $R_6$ we have two limit cycles: the inner cycle is stable, the outer cycle is unstable (Fig.7f). The outer cycle forms the boundary of the basin of attraction for the state $E_1$.

The diagram in Fig.6c is more complicated as compared to Fig.6a,b. In particular, a new region $R_8$ emerges, where three limit cycles can coexist: the inner limit cycle is stable, the middle cycle is unstable and the outer one is stable. The corresponding portrait is shown in Fig.7h.

For the Ivlev and trigonometric formulations of *h*(*y*), the bifurcation diagrams in the $\delta$–*m* plane constructed for $\alpha$ above and below the $BT^0$ point are topologically equivalent (see supplementary material SM3). However, for fixed $\beta$ and $\alpha$ the location of the bifurcation curves as well as the types of bifurcation (e.g., $BT^+$ versus $BT^-$ type of bifurcation) in the $\delta$–*m* plane may be substantially different, especially when comparing the Monod parametrisation with the other two functional forms. This indicates sensitivity of the model to the functional form of *h*(*y*). We explore the structural sensitivity in more detail in the next section.

Consider now variation of the fourth model parameter $\beta$. A decrease in $\beta$ results in a shift of the saddle-node and Hopf bifurcation surfaces in the $(\alpha ,\delta ,m)$ space closer to the ${\alpha }{-}{\delta }$ plane. The length of the curve of Bogdanov–Takens points (the intersection between the saddle-node and Hopf bifurcation) will be shortened and it moves upwards on the saddle-node bifurcation surface. The codimension 3 Bogdanov–Takens bifurcation is still observed. The above properties hold true for all three functional forms of *h*(*y*) considered.

Finally, we consider the case where $\beta$ gradually decreases and the other parameters are kept fixed. This corresponds to the ecologically important scenario in which the environment undergoes gradual eutrophication: an increase in the carrying capacity *K* in the original model (1) corresponds to a proportional decrease in $\beta$ in the dimensionless model. An example of a diagram in the $\beta {-}{m}$ plane is constructed for the Monod parametrisation of *h*(*y*) (Fig. 6d). The parameter regions in the diagram have the same meanings as in Fig. 6a-c. A gradual decrease of $\beta$ results in destabilisation of the coexistence equilibrium and a further collapse of the population of predator (transition from region $R_2$ to region $R_4$). Thus in a eutrophic environment, the only stable equilibrium in the model is $E_{1}$ being a predator free equilibrium. Note that in this diagram we have a new region denoted as $R_7$ in which a locally stable equilibrium $E_{1*}$ is surrounded by two limit cycles: the inner cycle is unstable whereas the outer one is stable. The corresponding phase portrait is shown in Fig. 7g. Similar bifurcation behavior is observed for the other two parameterisations of *h*(*y*).

## Structural sensitivity of the model

An important part of our investigation is exploring the dependence of model behaviour on the choice of parametrisation of the Allee effect given by *h*(*y*). In the previous section, we show that the skeleton of the bifurcation diagram is topologically robust to the mathematical shape of *h*(*y*) (Fig. 5) when we use three different parameterisations given by the Monod, Ivlev and hyperbolic tangent functions. The relative positions of bifurcation surfaces and possible dynamical regimes remain the same. On the other hand, we also find that for a fixed set of parameters the parametric diagrams can differ considerably, even for close functions *h*(*y*). This property is known as structural sensitivity of biological models.

**Fig. 8:**
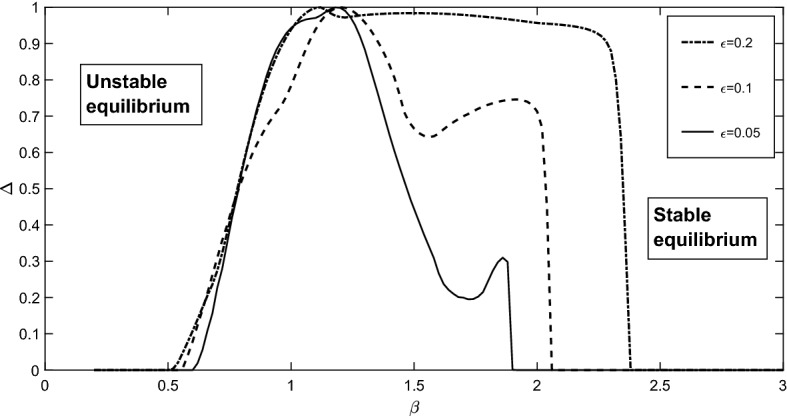
The degree of structural sensitivity $\Delta$ in model (2) to variation of the parameterisation of the Allee effect in the predator *h*(*y*), shown for different $\beta$. The $\epsilon$-neighbourhood of *h*(*y*) is defined using the relative difference between the base function and its perturbations. Destabilisation of the equilibrium for the base function (Monod parameterisation) occurs at $\beta =0.975$ For further detail see the text and also (Adamson and Morozov 2013, 2014a). The other parameters are $\alpha = 7.1$, $\delta =0.12$, $m=1.8$ and $D=10$

Structural sensitivity of models is an important issue in ecological modelling with a large number of insightful examples provided (Adamson and Morozov 2013, 2014a, b; Aldebert et al. 2019; Flora et al. 2011; Wood and Thomas 1999). The biological rationale behind this idea is that any realistic ecological dependence *h*(*y*) can be some combination of the three parameterisations considered above, or, more generally, any other mathematical functions. Here we explore the sensitivity of model predictions to a small variation of *h*(*y*) by considering the entire set of functions which satisfy assumptions (A1)–(A5), and we use the methodology from (Adamson and Morozov 2013, 2014a). Here we investigate the sensitivity of the stability of the interior non-saddle stationary state to small but finite perturbation of *h*(*y*) starting from one of the functional forms considered above. Mathematically, we consider the following situation.

Let the base function *h*(*y*) be of Monod type. We consider small deviations $h_1(y)$ from the base function *h*(*y*) such that $h(y)(1-\epsilon ) \le h_1(y)\le h(y)(1+\epsilon )$ and the second derivative of $h_1(y)$ is negative and bounded, $-D< h''_1(y) \le 0$, $D>0$ (note that the base function also satisfies this condition). For simplicity we slightly relax our assumption (A5) and allow $h_1(y) \rightarrow 1+\epsilon$, $y \rightarrow \infty$, although this fact is not crucial for the general outcome and conclusions. We conduct the sensitivity analysis in the same way as when investigating the role of the functional response of the predator in stability dynamics in the Rosenzweig–MacArthur model (Adamson and Morozov 2013). To quantify the structural sensitivity to the choice of $h_1(y)$ we use the degree of structural sensitivity $\Delta$ introduced in (Adamson and Morozov 2013). $\Delta$ represents the probability that for two randomly chosen functions of $h_1(y)$, the coexistence stationary state will have the same stability properties (Adamson and Morozov 2013), to be stable or unstable. The maximal degree of sensitivity possible in the system is equal to one, signifying maximal uncertainty in the system

Figure 8 shows the degree of sensitivity $\Delta$ as the parameter $\beta$ is varied. From the figure one can conclude that a decrease in $\beta$ eventually results in destabilization of the system for any parametrisation of $h_1(y)$: we gradually move from the region of stability to the region of instability shown in the figure. However, depending on the particular choice of $h_1(y)$, this destabilisation may occur within a wide range of $\beta$, thus the system has a plasticity to resist destabilisation caused, for example, by eutrophication (a high value of *K* in the original system signifies a low $\beta$ in the dimensionless model). Another important observation is that the system exhibits large uncertainty even if the deviation $\epsilon$ from the base function is small ($<5$%). Considering the Ivlev and the hyperbolic tangent as the base function provides similar results.

## Discussion

The role of the Allee effect in population dynamics has been largely addressed in both empirical and theoretical literature. Surprisingly enough, there has been almost no thorough mathematical investigation into the bifurcation structure of any predator–prey model with an Allee effect in the predator, in particular, this concerns the realistic scenario, where the Allee effect is included in the numerical response of the predator without affecting its functional response (Berec et al. 2007; Courchamp et al. 2008; Dennis 1989). This is in a striking contrast to the situation with single species population models or classical predator–prey models with an Allee effect in the prey growth, which have been discussed in all detail and are now included in standard student textbooks in mathematical ecology (Kot 2001). The current study is intended to partially bridge the existing gap. Importantly, our results are not based on a particular mathematical formulation of the function describing the Allee effect, rather we consider various parameterisations of *h*(*y*) which satisfy only few qualitative constraints (A1)–(A5). We have also addressed (for the first time) the issue regarding the sensitivity of the model with respect to parameterisation of the Allee effect (known as the structural sensitivity).

In this section, we will mostly focus on ecological implications of the mathematical results obtained in the previous sections. We should stress, however, that it is crucial to define the way of how we should formally assess the consequences of the Allee effect on the population dynamics. Indeed, this question is far to be a trivial one since distinct paradigms exist in the literature (Alves and Hilker 2017; Berec et al. 2007; Courchamp et al. 2008; Dennis 1989). In fact, evaluation of the role of the Allee effect in population success should largely depend on the choice of the initial density, and this fact is still somehow disregarded in the literature. Indeed, consider two populations, where one possesses a self-accelerating per capita reproduction rate, and the other one which is characterised by a constant per capita growth rate: for simplicity we assume the growth rates of both populations at some low density to be the same. Then an increase in the population density would result in an increase in the per capita growth rate of the species with an Allee effect, and this will clearly indicate the benefits of possessing an Allee effect. For example, hunting cooperation is considered to be beneficial for predators up to certain level of population density (Alves and Hilker 2017). On the other hand, considering higher population densities as the starting point for comparison (e.g. population densities where the Allee effect is not pronounced) will show a different outcome. For the same initial per capita growth rates, the species without an Allee effect will be more advantageous since a sudden drop of the population size would not largely affect its per capita growth whereas the population with an Allee effect may exhibit a significant decline in reproduction rate with a threat of extinction. For example, low fertilization efficiency, a lack of mating partners, and sperm limitation are usually considered as negative and undesirable features for population persistence (Berec et al. 2007; Courchamp et al. 2008; Dennis 1989).

Arguably, for many species, their reproduction rate is often empirically estimated at densities which are away from the extinction threshold, where the Allee effect is not well-pronounced. As such, we suggest that in theoretical models the impact of the Allee effect on dynamics should be assessed via comparison with a scenario without an Allee effect, where for both scenarios per capita reproduction rates are assumed to be the same at some ‘safe’ densities. For the current theoretical study, this mathematically signifies that we need to compare the Allee effect in model (2) with the same model with $h(y)\equiv 1$ since for large *y* the value of *h*(*y*) tends to unity, which corresponds to the classical Rosenzweig–MacArthur predator–prey model (Kot 2001). Note that the model with the Allee effect becomes the Rosenzweig–MacArthur model in the case $\delta \rightarrow 0+$.

Following the above philosophy, our first important conclusion is that introducing the Allee effect in predator’s numerical response generally acts as a destabilising factor of a stable coexistence of the prey and the predator in the case where the predator is specialist. Moreover, the Allee effect can result in extinction of predators regardless of initial density. This can be seen from the bifurcation diagrams, when the parameter $\delta$, characterising the strength of the Allee effect, increases from small values $\delta \ll 1$, corresponding to the Rosenzweig–MacArthur predator–prey model, to some large values. Interestingly, destabilising influence of the Allee effect in the predator is observed even for a linear functional response as well (see supplementary material SM4 for detailed illustration). It is well-known that the coexistence equilibrium of the classical Rosenzweig–MacArthur predator–prey model with a linear functional response and a constant *h*(*y*) is globally stable. Introducing an Allee effect into the predator growth results in stability loss, with either generating sustained oscillations (in this case both prey and predator still persist in the system in an oscillatory mode) or leading to extinction of predator (via different scenarios) for any initial population density. Destabilisation of the system is facilitated with a pronounced saturation in the functional response of the predator (small $\beta$) and with an increase in the strength of the Allee effect—interpreted in the model as a gradual increase in $\delta$. Biologically, this signifies that having density-dependent *h*(*y*) rather than a constant *h*(*y*) efficiency of predator impedes control over the prey population: the response of the predator to variation of the prey density in the system with the Allee effect becomes delayed and this allows the prey to escape from the control.

The predicted by the model destabilising role of the Allee effect demonstrates that cyclic population dynamics should occur in predator–prey systems more frequently as it was suggested earlier. For example, in the case of a Holling type I functional response, which is well-known to be stabilising, empirically observed oscillations of population numbers are usually attributed to factors as environmental/demographic noise, seasonal forcing, complex age structure of the population, complexity of the food webs, etc (see (Barraquand et al. 2017) and the references therein). On the other hand, an Allee effect can be an alternative explanation of oscillatory dynamics of a large number of case studies which occur in non-eutrophic environments with a stabilising functional response of the predator/consumer.

Our second important conclusion is that destabilisation of the coexistence equilibrium in the system with an Allee effect in the predator can occur not only via a supercritical Hopf bifurcation scenario (appearance of a small-amplitude stable limit cycle) but via a subcritical one. In the latter case, destabilisation of the equilibrium will leads to eventual extinction of the predator since the only possible attractor of the model is the state with only prey population being present (regime $R_4$ in the model, Fig. 7). On the other hand, eutrophication of the environment—which in the dimensionless model corresponds to a decrease in the parameter $\beta$—would eventually result in extinction of the predator regardless of the scenario of stability loss of the equilibrium (supercritical and subcritical). Indeed, in the case of a supercritical Hopf bifurcation, the resultant predator–prey cycle grows in size and enters the basin of attraction of the prey-only equilibrium: mathematically this occurs via a homoclinical bifurcation.

Our results may have important implications for the biological control of the pests by predators and parasitoids. It has been reported that in a large number of cases, biological control agents have failed to get established even if under laboratory conditions they could survive by consuming target pest species (Bellows 2001; Roderick and Navajas 2003; Orr 2009; Bompard et al. 2013). A possible explanation is the presence of an Allee effect in the biological control agents which becomes more pronounced in the environment as in a lab. For example, the initial density of the predator can quickly fall below the critical threshold because of dispersal and diffusion. However, there can be a more complicated scenario where the initial density of the predator can be very high, but a pronounced Allee effect will still not allow a long-term persistence of species. Our model mathematically describes this as a globally unstable co-existence state (regime $R_4$ in the model, Fig. 7). As a conclusion, the choice of the appropriate species for an efficient biological control should be made carefully. For example, for parasitoids it is preferred to use haplodiploid species (i.e., where the males are haploid and females diploid) to alleviate the negative demographic consequences of mate-finding Allee effects, which is well-pronounced in diploid species (Hopper and Roush 1993; Bompard et al. 2013).

Thirdly, we found that inclusion of the Allee effect in the predator growth largely increases the complexity of the system as compared to the scenario without an Allee effect, i.e., the original Rosenzweig–MacArthur predator–prey model. One of the most interesting observation is the possibility of non-trivial multiple attractors, which can be interpreted as alternative ecosystem states. The model predicts two such regimes denoted by $R_7$ and $R_8$, see Fig. 7. They are (i) a stable equilibrium coexisting with a stable limit cycle and (ii) two stable limit cycles, respectively. Note that in the literature, there is an ongoing debate on possible origins of alternative states generated through various ecological mechanisms (Schröder et al. 2005). In the simplest case, alternative stationary states are two contrasting equilibria (Scheffer et al. 2003; Scheffer and Carpenter 2003). However, more interesting patterns with non-equilibrium coexisting attractors have been reported in the literature as well. In particular, empirical observations demonstrate the possibility of alternative attractors where depending on initial condition the population dynamics can show either cyclic or stable coexistence scenario (Zamamiri et al. 2001). Other empirical studies demonstrate the possibility of coexisting cyclic oscillations with contrasting amplitude and periodicity (McCauley et al. 1999; Henson et al. 2002). Interestingly, in the Daphnia-algae predator–prey system reported in (McCauley et al. 1999), the observed coexisting cycles were considered to be a consequence of variability of the available food for zooplankton (e.g. a more complicated prey-dependent functional response of the predator or food-dependent efficiency of reproduction), whereas the density dependence of Daphnia’s vital rates was somewhat intentionally disregarded. On the other hand, the presence of an Allee effect (e.g. an emergent Allee effect is known to be present in Daphnia (de Roos et al. 2003)) can be arguably an alternative explanation of the co-existing cyclic behaviour.

Finally, we should stress that unlike the scenario with the Allee effect in prey—which is currently considered to be straightforward in the literature—including the Allee effect in predators can be somewhat tricky since this can be done either by modifying the functional response or the numeric response of the predator. Biologically, this signifies that distinct mechanisms of emerging the resultant demographic Allee effect—a decrease in reproduction at low population numbers—should be modelled in a different way. In particular, the mechanisms such as low fertilization efficiency, a lack of mating partners, sperm limitation, cooperative breeding and similar mechanisms (see empirical examples in Sect. 2) should be included in the numerical response of predator only, whereas collective exploitation of a resource such as cooperating hunting should be included in both functional and the numerical responses. It is rather surprising that this fact has not been largely explored in the literature yet.

As a first step to fill the existing gap, we compared the stability of two similar predator–prey systems: in one model the Allee effect was due to collective exploitation of resources (as in the study (Alves and Hilker 2017)) and in the other one the Allee effect was due to the lack of mating partners (for details see SM4). Note that unlike the mentioned work (Alves and Hilker 2017) we included saturation in the Allee effect in the functional response of predator (see the model equations in SM4). For simplicity, we considered the case where the functional response of the predator is of Holling type I. We found that a pronounced Allee effect (large saturation in *h*(*y*)) has somewhat different consequences for the two systems. Using bifurcation diagrams, we compared the stability regions for both models. We found that an Allee effect due to collective exploitation of resources considered in (Alves and Hilker 2017) facilitates persistence of the predator as compared to the scenario where the Allee effect is included only in numerical response (e.g. due to mate-finding) since the stability region for letter model is smaller in size. However, a more detailed comparison of the two mentioned approaches to modelling the Allee effect in predator should be an interesting separate study.

## Supplementary Information

Below is the link to the electronic supplementary material.

Supplementary material 1 (pdf 572 KB)
