# Supplementary material for: Bifurcation analysis of the predator–prey model with the Allee effect in the predator
Source: J Math Biol. 2021 Dec 30;84(1-2):7. doi: 10.1007/s00285-021-01707-x (PMC8718388; doi:10.1007/s00285-021-01707-x)
Supplement: Supplementary file 1 — Supplementary material 1 (pdf 572 KB) [file 285_2021_1707_MOESM1_ESM.pdf]

Bifurcation analysis of the predator-prey model with the Allee effect in the predator

Deeptajyoti Sen[1], Saktipada Ghorai[2], Malay Banerjee[2], Andrew Morozov[3,4]
[1] IISER Mohali, India
[2] IIT Kanpur, India
[3] University of Leicester, UK
[4] Severtsov Institute of Ecology and Evolution, Moscow, Russia

Supplementary material 1

Proof of Proposition 2: Suppose that the interior equilibrium undergoes a generalised Hopf (GH) bifurcation at $\hat{E} = (\hat{x}, \hat{y})$ for the parameter threshold (β_{GH}, m_{GH}). Since $\hat{E}$ is a non-trivial equilibrium point then it must satisfies (3). The Jacobian matrix at $\hat{E}$ is given by

$$J(\hat{E}) = \begin{bmatrix} \frac{\hat{x}}{\beta+\hat{x}}(1-\beta-2\hat{x}) & -\frac{\hat{x}}{\beta+\hat{x}} \\ \frac{\alpha\beta\hat{y}h(\hat{y})}{(\beta+\hat{x})^2} & \frac{\alpha\hat{x}\hat{y}}{\beta+\hat{x}}h'(\hat{y}) \end{bmatrix}.$$

Now using the condition (GH2), we find from the above Jacobian

$$\beta_{GH} = 1 - 2\hat{x} + \alpha\hat{y}h'(\hat{y}).$$

Now to find the first Lyapunov number at $\hat{E}$, we translate $\hat{E}$ to origin by using the transformation $u = x - \hat{x}$, $v = y - \hat{y}$ and we get

$$\dot{u} = au + bv + P(u, v),$$

$$\dot{v} = cu + dv + Q(u, v),$$

where $a = \frac{\partial F_1}{\partial x}\big|_{\hat{E}}$, $b = \frac{\partial F_1}{\partial y}\Big|_{\hat{E}}$, $c = \frac{\partial F_2}{\partial x}\big|_{\hat{E}}$, $d = \frac{\partial F_2}{\partial y}\Big|_{\hat{E}}$, $P(u, v)$ and $Q(u, v)$ are analytic function is given by

$$P(u, v) = \sum_{i+j\geq 2} a_{ij}u^i v^j,$$

$$Q(u, v) = \sum_{i+j\geq 2} b_{ij}u^i v^j,$$

where a_{ij} and b_{ij} are obtained from the following relations

$$a_{ij} = \frac{1}{i!j!}\frac{\partial^{i+j}F_1}{\partial x^i \partial y^j}\bigg|_{\hat{E}}, \quad b_{ij} = \frac{1}{i!j!}\frac{\partial^{i+j}F_2}{\partial x^i \partial y^j}\bigg|_{\hat{E}}.$$

Now first Lyapunov number is defined by

$$l = -\frac{3\pi}{2b\Delta^{\frac{3}{2}}}\left[\{ac(a_{11}^2 + a_{11}b_{02} + a_{02}b_{11}) + ab(b_{11}^2 + a_{20}b_{11} + a_{11}b_{02} - 2acb_{02}^2\right.$$
$$-2ab(a_{20}^2 - b_{20}b_{02}) - b^2(2a_{20}b_{20} + b_{11}b_{20}) + (bc - 2a^2)(b_{11}b_{02} - a_{11}a_{20})\}$$
$$\left.-(a^2 + bc)\{3(cb_{03} - a_{30}) + 2a(a_{21} + b_{12}) + (ca_{12} - bb_{21})\}\right].$$

We calculate the coefficients a_{ij}, b_{ij} and a, b, c, d of the first Lyapunov number at $\hat{E}$:

$$a = \frac{\hat{x}}{\beta + \hat{x}}(1 - \beta - 2\hat{x}), \quad b = -\frac{\hat{x}}{\beta + \hat{x}}, \quad c = \frac{\alpha\beta\hat{y}h(\hat{y})}{(\beta + \hat{x})^2}, \quad d = \frac{\alpha\hat{x}\hat{y}h^{'}(\hat{y})}{\beta + \hat{x}},$$

$$a_{20} = \left(\frac{\beta\hat{y}}{(\beta + \hat{x})^3} - 1\right), \quad a_{11} = -\frac{\beta}{(\beta + \hat{x})^2}, \quad a_{02} = 0,$$

$$b_{20} = -\frac{\alpha\beta\hat{y}h(\hat{y})}{(\beta + \hat{x})^3}, \quad b_{11} = \frac{\alpha\beta}{(\beta + \hat{x})^2}\left[h(\hat{y}) + \hat{y}h^{'}(\hat{y})\right], \quad b_{02} = \frac{\alpha\hat{x}}{2(\beta + \hat{x})}\left[2h^{'}(\hat{y}) + \hat{y}h^{''}(\hat{y})\right],$$

$$a_{30} = -\frac{\beta\hat{y}}{2(\beta + \hat{x})^4}, \quad a_{21} = \frac{\beta}{(\beta + \hat{x})^3}, \quad a_{12} = 0, \quad a_{03} = 0,$$

$$b_{30} = \frac{\alpha\beta\hat{y}h(\hat{y})}{(\beta + \hat{x})^4}, \quad b_{21} = -\frac{\alpha\beta}{(\beta + \hat{x})^3}\left[h(\hat{y}) + \hat{y}h^{'}(\hat{y})\right],$$

$$b_{12} = \frac{\alpha\beta}{2(\beta + \hat{x})^2}\left[2h^{'}(\hat{y}) + \hat{y}h^{''}(\hat{y})\right], \quad b_{03} = \frac{\alpha\hat{x}}{6(\beta + \hat{x})}\left[3h^{''}(\hat{y}) + \hat{y}h^{'''}(\hat{y})\right].$$

After some algebraic calculation we get (assuming that $h(y)$ is as smooth as that $h^{'''}(y)$ exist)

$$l(\hat{E}; \beta = \beta_{GH}, m = m_{GH})) = -\frac{3\Pi}{2b\Delta_{GH}^{\frac{3}{2}}}\left[Am_{GH}^2 + Bm_{GH} + C\right],$$

where

$$A = -\frac{\alpha\beta^2 m^2 y}{(\beta + x)^4}\left[2h^{'}(y) + \frac{y}{2}h^{''}(y)\right]\Big|_{(\hat{E};\beta = \beta_{GH}, m = m_{GH})},$$

$$B = \left[\frac{\beta(1 - \beta - 2x)}{(\beta + x)^3}\{\frac{\beta^2 y}{(\beta + x)^3} - \frac{2\alpha\beta xyh^{'}(y)}{(\beta + x)^2} - \frac{x}{\beta + x}(\frac{\beta y}{(\beta + x)^3} - 1)\}\right.$$
$$-\frac{\alpha\beta x}{(\beta + x)^3}(2h^{'}(y) + yh^{''}(y))\{\frac{\beta y(1 - \beta - 2x)}{2(\beta + x)^2} + \frac{\alpha xy}{2}(1 - \beta - 2x)$$
$$(2h^{'}(y) + h^{''}(y)) + \frac{xy}{(\beta + x)^2}(1 - \beta - 2x) - \frac{\alpha\beta y^2 h^{''}(y)}{2(\beta + x)^3} - \frac{2x(1 - \beta - 2x)}{\beta + x}\}$$
$$\left.-\frac{\beta x}{(\beta + x)^4}\{x(1 - \beta - 2x)^2 - \frac{\alpha\beta yh(y)}{\beta + x}\}\{\frac{\alpha y}{2}(3h^{''}(y) + yh^{'''}(y)) - \frac{1}{\beta + x}\}\right]\Big|_{(\hat{E};\beta = \beta_{GH}, m = m_{GH})},$$

$$
\begin{aligned}
C &= -\frac{x^2}{(\beta+x)^2}(1-\beta-2x)\left[\frac{\alpha^2\beta^2y^2(h(y))^2}{(\beta+x)^4}+\frac{\alpha\beta y h'(y)}{(\beta+x)^2}\left(\frac{\beta y}{(\beta+x)^3}-1\right)-\frac{\alpha\beta x}{2(\beta+x)^3}\right.\\
&\quad (2h'(y)+yh''(y))+2\left(\frac{\beta y}{(\beta+x)^3}-1\right)^2+\frac{2\alpha^2\beta xy(1-\beta-2x)h'(y)}{(\beta+x)^3}-\frac{x}{(\beta+x)^5}\\
&\quad \{x(1-\beta-2x)^2-\frac{\alpha\beta y h(y)}{\beta+x}\}\{-\frac{3\beta xy}{(\beta+x)^2}\\
&\quad \left.\left.+2\beta x(1-\beta-2x)(\frac{1}{\beta+x}+\frac{\alpha}{2}(2h'(y)+yh''(y)))-\frac{\alpha\beta xyh'(y)}{\beta+x}\}\right]\right|_{(\hat{E};\beta=\beta_{GH},m=m_{GH})}.
\end{aligned}
$$

Due to algebraic complexity we are unable to show that $l((\hat{E};\beta=\beta_{GH},m=m_{GH}))=0$, however it can be verified numerically. Thus we can conclude that the system (2) admits GH bifurcation for the parameter threshold $(\beta=\beta_{GH},m=m_{GH})$ at $\hat{E}$.

Supplementary material 2

Proof of Proposition 3: The conditions (BT1) and (BT2) are equivalent to the following ones

$$
\bar{x}h'(\bar{y})(1-\beta-2\bar{x})+\left.\frac{\beta h(\bar{y})}{\beta+\bar{x}}\right|_{(\beta_{BT},m_{BT})} = 0,
$$

and

$$
1-\beta-2\bar{x}+\alpha\bar{y}h'(\bar{y})\Big|_{(\beta_{BT},m_{BT})} = 0.
$$

It is difficult to find the explicit expressions of the bifurcation parameters for the BT bifurcation thresholds since the above equations contain unknown function $h(y)$ and the coordinates of the equilibrium points contain the parameters implicitly. However, we can define the bifurcation thresholds as follows:

$$
\begin{aligned}
\beta_{BT} &= 1-2\bar{x}+\alpha\bar{y}h'(\bar{y}),\\
m_{BT} &= \alpha\left[\bar{x}h'(\bar{y})(1-\beta_{BT}-2\bar{x})+h(\bar{y})\right].
\end{aligned}
$$

Let us consider the small perturbation around the bifurcation threshold (β_{BT},m_{BT}) by $(\beta_{BT}+\lambda_1,m_{BT}+\lambda_2)$, where λ_i, $i=1,2$ are sufficiently small, and substituting it into the system we get

$$
\begin{aligned}
\frac{dx}{dt} &= x(1-x)-\frac{xy}{\beta_{BT}+\lambda_1+x} \equiv F_1(x,y,\lambda_1),\\
\frac{dy}{dt} &= \frac{\alpha xy}{\beta_{BT}+\lambda_1+x}h(y)-(m_{BT}+\lambda_2)\,y \equiv F_2(x,y,\lambda_1,\lambda_2),
\end{aligned}
$$

Now we shift the equilibrium $\bar{E}=(\bar{x},\bar{y})$ to the origin by the coordinate transform $X=x-\bar{x}$, $Y=y-\bar{y}$ and substitute it in model (2) to obtain

$$
\begin{aligned}
\dot{X} &= F_{10}+\tilde{a}X+\tilde{b}Y+p_{20}X^2+p_{11}XY+H_1(X,Y),\\
\dot{Y} &= F_{20}+\tilde{c}X+\tilde{d}Y+q_{20}X^2+q_{11}XY+q_{02}Y^2+H_2(X,Y),
\end{aligned}
$$

where

$$
\begin{aligned}
F_{10} &= \bar{x}\,(1-\bar{x}) - \frac{\bar{x}\bar{y}}{\beta_{BT}+\lambda_1+\bar{x}}, \quad \tilde{a} \;=\; 1-2\bar{x} - \frac{\bar{y}}{\beta_{BT}+\lambda_1+x} + \frac{\bar{x}\bar{y}}{(\beta_{BT}+\lambda_1+x)^2}, \\
\tilde{b} &= -\frac{\bar{x}}{\beta_{BT}+\lambda_1+\bar{x}}, \quad p_{20} \;=\; -\left(1 - \frac{\bar{y}}{\beta_{BT}+\lambda_1+\bar{x}} + \frac{\bar{x}\bar{y}}{(\beta_{BT}+\lambda_1+\bar{x})^2}\right), \\
p_{11} &= -\frac{\beta_{BT}+\lambda_1}{(\beta_{BT}+\lambda_1+\bar{x})^2}, \quad F_{20} \;=\; \frac{\alpha\bar{x}\bar{y}}{\beta_{BT}+\lambda_1+\bar{x}}h(\bar{y}) - (m+\lambda_2\bar{y}), \\
\tilde{c} &= \frac{\alpha(\beta_{BT}+\lambda_1)\bar{y}h(\bar{y})}{(\beta_{BT}+\lambda_1+\bar{x})^2}, \quad \tilde{d} \;=\; \frac{\alpha\bar{x}}{\beta_{BT}+\lambda_1+\bar{x}}\Big(h(\bar{y})+\bar{y}h^{'}(\bar{y})\Big) - (m_{BT}+\lambda_2), \\
q_{20} &= -\frac{\alpha(\beta_{BT}+\lambda_1)\bar{y}h(\bar{y})}{(\beta_{BT}+\lambda_1+\bar{x})^3}, \quad q_{11} \;=\; \frac{\alpha(\beta_{BT}+\lambda_1)}{(\beta_{BT}+\lambda_1+\bar{x})^2}\Big[h(\bar{y})+\bar{y}h^{'}(\bar{y})\Big], \\
q_{02} &= \frac{\alpha\bar{x}}{\beta_{BT}+\lambda_1+\bar{x}}\Big[2h^{'}(\bar{y})+\bar{y}h^{''}(\bar{y})\Big],
\end{aligned}
$$

and H_1, H_2 are power series of X and Y with terms X^iY^j, $i+j \geq 3$. Now by using affine transformation $X_1 = X$, $X_2 = \tilde{a}X + \tilde{b}Y$, the system transferred to

$$
\begin{aligned}
\dot{X}_1 &= F_{10} + X_2 + l_{20}X_1^2 + l_{11}X_1X_2 + \tilde{H}_1(X_1,X_2), \\
\dot{X}_2 &= k_0 + k_{10}X_1 + k_{01}X_2 + k_{20}X_1^2 + k_{11}X_1X_2 + k_{02}X_2^2 + \tilde{H}_2(X_1,X_2),
\end{aligned}
$$

where,

$$
\begin{aligned}
l_{20} &= p_{20} - \frac{\tilde{a}}{\tilde{b}}p_{11}, \quad l_{11} \;=\; \frac{p_{11}}{\tilde{b}}, \\
k_0 &= \tilde{a}F_{10} + \tilde{b}F_{20}, \quad k_{10} \;=\; \tilde{b}\tilde{c} - \tilde{a}\tilde{d}, \quad k_{01} \;=\; \tilde{a} + \tilde{d}, \\
k_{20} &= \tilde{a}p_{20} - \frac{\tilde{a}}{\tilde{b}}p_{11} + \tilde{b}q_{20} - \tilde{a}q_{11} + \frac{\tilde{a}^2}{\tilde{b}}q_{02}, \\
k_{11} &= \frac{\tilde{a}}{\tilde{b}}p_{11} + q_{11} - 2\frac{\tilde{a}}{\tilde{b}}q_{02}, \quad k_{02} \;=\; \frac{q_{02}}{\tilde{b}},
\end{aligned}
$$

and $\tilde{H}_1$, $\tilde{H}_2$ are power series of X_1 and X_2 with terms $X_1^iX_2^j$, $i+j \geq 3$. Now using the C^∞ change of coordinates in the neighbourhood of origin by the following transformation

$$
v_1 \;=\; X_1 - \frac{l_{11}+k_{02}}{2}X_1^2, \quad v_2 \;=\; X_2 + l_{20}X_1^2 - k_{02}X_1X_2,
$$

the system reduces to

$$
\begin{aligned}
\dot{v}_1 &= F_{10} + s_{10}v_1 + v_2 + s_{20}v_1^2 + R_1(v_1,v_2), \\
\dot{X}_2 &= k_0 + t_{10}v_1 + t_{01}v_2 + t_{20}v_1^2 + t_{11}v_1v_2 + R_2(v_1,v_2),
\end{aligned}
$$

where

$$\begin{aligned}
s_{10} &= -\frac{F_{10}}{\tilde{b}}(p_{11}+q_{02}), \quad s_{20} = -\frac{F_{10}}{2\tilde{b}^2}(p_{11}+q_{02})^2,\\
t_{10} &= k_{10}+2F_{10}l_{20}-k_0k_{02}, \quad t_{01} = k_{01}-\frac{F_{10}}{\tilde{b}}q_{02},\\
t_{20} &= \frac{k_{10}}{2}(l_{11}+k_{02})-k_{01}l_{20}+k_{20}+F_{10}l_{20}(l_{11}+k_{02})-k_{01}k_{02}-\frac{k_0k_{02}}{2}(l_{11}+k_{02})+F_{10}l_{20}k_{02},\\
t_{11} &= k_{01}k_{02}+k_{11}+2l_{20}-k_{01}k_{02}-k_{02}^2F_{10},
\end{aligned}$$

and R_1, R_2 are power series of v_1 and v_2 with terms $v_1^iv_2^j$, $i+j\geq 3$.

Now again using the C^∞ change of coordinates in the neighbourhood of origin by the following transformation

$$z_1 = v_1, \quad z_2 = F_{10}+s_{10}v_1+v_2+s_{20}v_1^2,$$

the system becomes reduced to

$$\begin{aligned}
\dot{z}_1 &= z_2+\tilde{R}_1(z_1,z_2),\\
\dot{z}_2 &= w_0+w_{10}z_1+w_{01}z_2+w_{20}z_1^2+w_{11}z_1z_2+\tilde{R}_2(z_1,z_2),
\end{aligned}$$

where

$$\begin{aligned}
w_0 &= t_0-t_{01}F_{10}, \quad w_{10} = t_{10}-t_{01}s_{10}-t_{11}F_0, \quad w_{01} = s_{10}+t_{01},\\
w_{20} &= t_{20}-t_{01}s_{20}-t_{11}s_{10}, \quad w_{11} = t_{11}+2s_{20},
\end{aligned}$$

and $\tilde{R}_1$, $\tilde{R}_2$ are power series of z_1 and z_2 with terms $z_1^iz_2^j$, $i+j\geq 3$. Finally, using the C^∞ change of coordinates in the neighbourhood of origin by the following transformation

$$n_1 = z_1, \quad n_2 = z_2+\tilde{R}_1(z_1,z_2),$$

the system changes to

$$\begin{aligned}
\dot{n}_1 &= n_2,\\
\dot{n}_2 &= P(n_1,\lambda_1,\lambda_2)+n_2\phi(n_1,\lambda_1,\lambda_2)+n_2^2\xi(n_1,n_2,\lambda_1,\lambda_2),
\end{aligned}$$

with

$$\begin{aligned}
P(n_1,\lambda_1,\lambda_2) &= w_0+w_{10}n_1+w_{20}n_1^2+P_1(n_1),\\
\phi(n_1,\lambda_1,\lambda_2) &= w_{01}+w_{11}n_1+\phi_1(n_1),\\
\xi(n_1,n_2,\lambda_1,\lambda_2) &= \xi_1(n_1,n_2).
\end{aligned}$$

Here P_1, ϕ_1, ξ_1 are the power series in terms of n_1^i, $i\geq 3$, n_1^i, $i\geq 2$ and $n_1^in_2^j$, $i+j\geq 1$ respectively. Now applying Malgrange preparation theorem to the function P, we get

$$P(n_1,\lambda_1,\lambda_2) = \left(n_1^2+\frac{w_{10}}{w_{20}}n_1+\frac{w_0}{w_{20}}\right)B(n_1,\lambda_1,\lambda_2),$$

where B is a power series of n_1 and $B(0, \lambda_1, \lambda_2) = w_{20}$. Now using the transformation $u_1 = n_1$, $u_2 = \frac{n_2}{\sqrt{B(n_1, \lambda_1, \lambda_2)}}$ and $T = \int_0^t \sqrt{B(n_1(s), \lambda_1, \lambda_2)}ds$, the system reduces to

$$\dot{u}_1 = u_2,$$

$$\dot{u}_2 = \frac{w_0}{w_{20}} + \frac{w_{10}}{w_{20}}u_1 + \frac{w_{01}}{\sqrt{w_{20}}}u_2 + u_1^2 + \frac{w_{11}}{\sqrt{w_{20}}}u_1u_2 + Q_1(u1, u_2, \lambda_1, \lambda_2),$$

where Q_1 is a power series in (u_1, u_2) with power $u_1^i u_2^j$, $i + j \geq 3$ and $j \geq 2$. Now applying the final transformation

$$y_1 = u_1 + \frac{w_{10}}{2w_{20}}, \quad y_2 = u_2,$$

the system transforms to

$$\dot{y}_1 = y_2,$$

$$\dot{y}_2 = \mu_1(\lambda_1, \lambda_2) + \mu_2(\lambda_1, \lambda_2)y_2 + y_1^2 + \frac{w_{11}}{\sqrt{w_{20}}} + Q_2(u1, u_2, \lambda_1, \lambda_2),$$

where

$$\mu_1(\lambda_1, \lambda_2) = \frac{w_0}{w_{20}} - \left(\frac{w_{10}}{2w_{20}}\right)^2,$$

$$\mu_2(\lambda_1, \lambda_2) = \frac{w_{01}}{\sqrt{w_{20}}} - \frac{w_{10}w_{11}}{2w_{20}\sqrt{w_{20}}},$$

and Q_2 is a power series in y_1 and y_2 of the form $y_1^i y_2^j$, $i + j \geq 3$.

To check the non-degeneracy conditions for BT bifurcation at $\bar{E}$ we need to check the signs of k_{20} and $l_{20} + k_{11}$ for $\lambda_1 = 0$ and $\lambda_2 = 0$ i.e. at $\beta = \beta_{BT}$ and $m = m_{BT}$. Now

$$\begin{aligned} k_{20} &= aa_{20} - \frac{a^2}{b}a_{11} + bb_{20} - ab_{11} + \frac{a^2}{b} \\ &= \Bigg[-\frac{\bar{x}^2}{(\beta_{BT} + \bar{x})^2}(1 - \beta_{BT} - 2\bar{x}) + \frac{\alpha\beta_{BT}\bar{x}h(\bar{y})}{(\beta_{BT} + \bar{x})^2} - \frac{\alpha\bar{x}}{(\beta_{BT} + \bar{x})^2}(1 - \beta_{BT} - 2\bar{x}) \\ &\quad h'(\bar{y})\left\{\beta_{BT}(1 - \bar{x}) - 2\bar{x}(1 - \beta_{BT} - 2\bar{x}) + \bar{x}\bar{y}(1 - \beta_{BT} - 2\bar{x})h''(\bar{y})\right\}\Bigg], \end{aligned}$$

where a, b, c, d, a_{ij}, b_{ij}, $i + j \geq 1$ are same as in the Supplementary Material A except one needs to calculate the coefficients at $\bar{E}$. We have $1 - \beta_{BT} - 2\bar{x} < 0$, as α and $h(y)$ is positive. Using this with the condition (A4) we can find $k_{20} > 0$

$$l_{20} = a_{20} - \frac{a}{b}a_{11} = -\frac{\bar{x}}{\beta_{BT} + \bar{x}} < 0$$

and

$$\begin{aligned} k_{11} &= \frac{a}{b}a_{11} + b_{11} - \frac{2a}{b}b_{02} \\ &= (1 - \beta_{BT} - 2\bar{x})\left[\frac{\beta_{BT}}{(\beta_{BT} + \bar{x})^2} + \alpha\bar{x}(1 - \bar{x})h''(\bar{y})\right] + \alpha(1 - 2\bar{x})h'(\bar{y}). \end{aligned}$$

Now sign(k_{11}) may changes from negative to positive or vice versa. Therefore $l_{20} + k_{11}$ may or may not be zero. If $l_{20} + k_{11} \neq 0$ then the system near the BT bifurcation point is topologically equivalent to the following system

$$\begin{aligned}\dot{x}_1 &= x_2,\\ \dot{x}_2 &= \mu_1 + \mu_2 x_2 + x_1^2 \pm x_1 x_2,\end{aligned}$$

which is the normal form of the BT bifurcation of co-dimension two. The coefficient in front of x_1x_2 can be either $+1$ or -1 according as sign$(k_{20}(l_{20} + k_{11}))$. If the sign is positive then the predator-prey model undergoes a subcritical BT bifurcation at $\bar{E}$ and if the sign is negative then the model undergoes supercritical BT bifurcation at $\bar{E}$.

In the case where $l_{20} + k_{11} = 0$, we have a co-dimension 3 Bogdanov-Takens bifurcation and the system is topologically equivalent to the following one

$$\begin{aligned}\dot{x}_1 &= x_2,\\ \dot{x}_2 &= \mu_1 + \mu_2 x_2 + \mu_3 x_1 x_2 + x_1^2 \pm x_1^3 x_2.\end{aligned}$$

Note that this type of co-dimension 3 Bogdanov-Takens bifurcation includes a double-equilibrium point.

Supplementary material 3

Here we resent several α cross sections of the (α, δ, m) bifurcation diagrams shown in Fig. 5 of the main text. We consider the case where parametrization of $h(y)$ is given either the Ivlev or the trigonometric response.

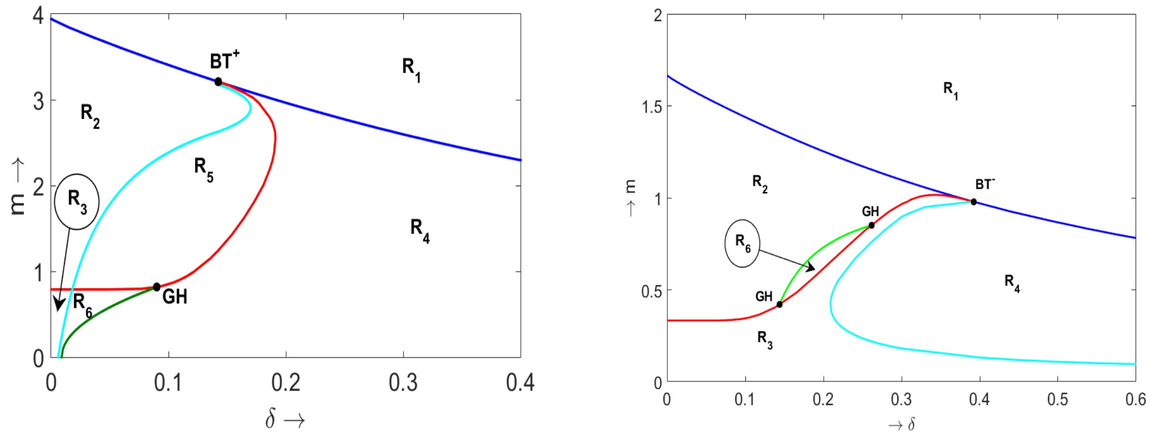

(a) (b)

Figure 1: S. Bifurcation diagrams in $\delta - m$ parametric plane for the Ivlev parametrization of Allee effect function. Parameter values are (a) $\alpha = 7.1$, $\beta = 0.8$; (b) $\alpha = 3$, $\beta = 0.8$. The dark blue curve is saddle-node bifurcation curve, the red curve is a Hopf bifurcation curve, the green curve is the curve of a saddle-node bifurcation of limit cycles. The cyan curve describes a homoclinic bifurcation.

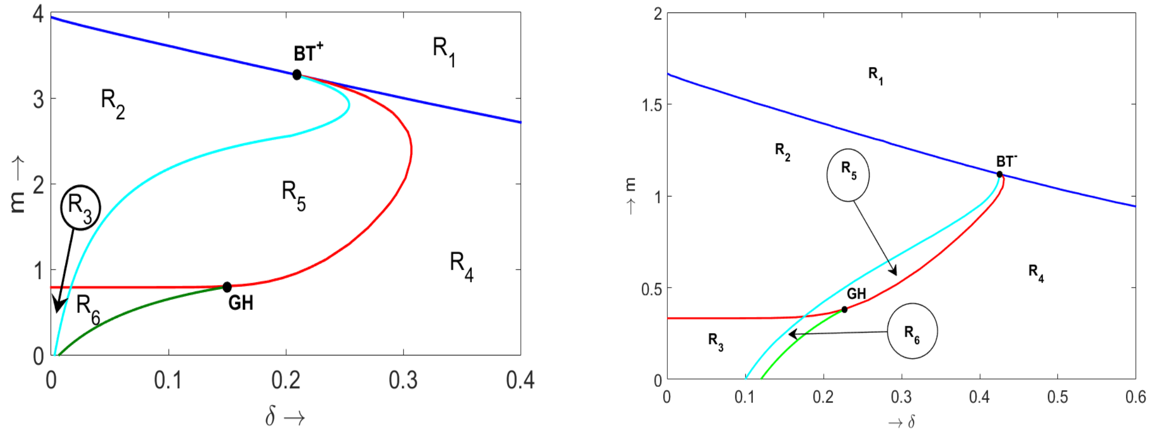

(a) (b)

Figure 2: S. Bifurcation diagrams in $\delta - m$ parametric plane for the tan-hyperbolic parametrization of $h(y)$. Parameter values are (a) $\alpha = 7.1$, $\beta = 0.8$; (b) $\alpha = 3$, $\beta = 0.8$. The dark blue curve is saddle-node bifurcation curve, the red curve is a Hopf bifurcation curve, the green curve is the curve of a saddle-node bifurcation of limit cycles. The cyan curve describes a homoclinic bifurcation.

Firstly, we present the bifurcation diagrams for the model with the Ivlev response constructed for $\alpha = 7.1$ and $\alpha = 3$ which is shown in Fig. 1S. The bifurcation diagrams contain saddle-node bifurcation curve (blue), Hopf-bifurcation curve (red) and two global bifurcation curves such as the homoclinic bifurcation curve (cyan) and the saddle-node bifurcation of limit cycles (green). Two local bifurcation curves intersect at the Bogdanov-Takens(BT) bifurcation point and generalised Hopf bifurcation (GH) points are located on the Hopf bifurcation curve. The homoclinic bifurcation curve and the saddle-node bifurcation curve of limit cycles emerge from the BT and GH points, respectively. Note that for $\alpha = 3$, we find two GH points which are connected by a global bifurcation curve. These four curves divide the parametric domain into six and five regions R_j respectively. Regions are described in detail in the main text.

Secondly, we present the bifurcation diagrams constructed for the hyperbolic tangent parametrisation of $h(y)$ which are shown in Figs. 2S. We consider the same values of the parameter α. The meaning of the curves and domains are the same as in the previous figure as well as in the main text. One can see that although the mutual position and the shape of bifurcation curves are similar to the Ivlev parameterisations, their shape is slightly different as those in Figs. 1S.

Supplementary Material 4

Here we will discuss the dynamics of two prey-predator models consisting of Allee effect in the predator: one with the Allee function in both functional and numerical responses (System (13)) and another with the Allee function in numerical response only (System (14)). First one describes the Allee effect due to collective exploitation of resources and another describes the same due to the lack of mating partner. We consider RM model with linear functional response and choose Monod parametrisation ($h(y) = \frac{y}{y+\delta}$) for the Allee function. Without loss of generality, we consider dimensionless model and the evolving equations are in the following:

$$\dot{x} = x(1-x) - \frac{xy^2}{y+\delta}$$
$$\dot{y} = \frac{\alpha x y^2}{y+\delta} - my \tag{13}$$

$$\dot{x} = x(1-x) - xy$$
$$\dot{y} = \frac{\alpha x y^2}{y+\delta} - my \tag{14}$$

To illustrate the dynamics of the above models, we construct bifurcation diagrams for both models in δ-m parametric plane for fixed $\alpha = 8$. Fig. 3S present the bifurcation portraits of the model (13) consisting three local bifurcation curves: one saddle-node bifurcation curve (blue), two Hopf-bifurcation curves (red) and two non-local bifurcation curves, known as homoclinic bifurcation (cyan). Also the bifurcation diagram consists two BT points, corresponds to Bogdanov-Takens bifurcation of co-dimension 2, where Hopf-bifurcation curve meets tangentially to the saddle-node bifurcation curve and a global bifurcation curve emerges from it. These curves divide the parametric plane into 5 regions ($R_1 - R_5$) with one common region (R_2). We label this regions as the same way we did for other bifurcation portraits. Secondly we construct the bifurcation diagram of the model (14) in Fig. 4S when Allee function occurs in the numerical response only. This diagram consists same number of local and global bifurcation curves as previous bifurcation diagram (Fig. 3S), rather these diagrams looks topologically equivalent to each other.

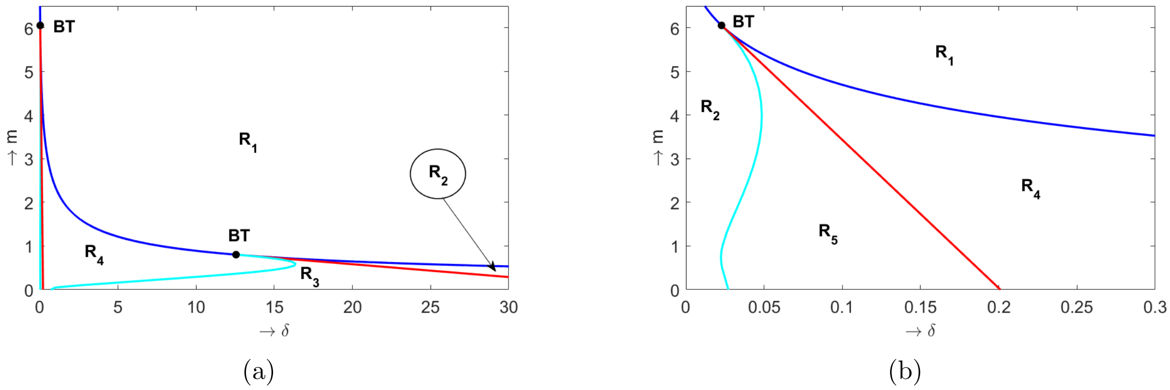

Figure 3: S. (a) Two-parametric bifurcation diagram of the system (13) in δ-m parametric plane at $\alpha = 8$, (b) zoomed version (a) near origin. Description of all the curves are given in the text.

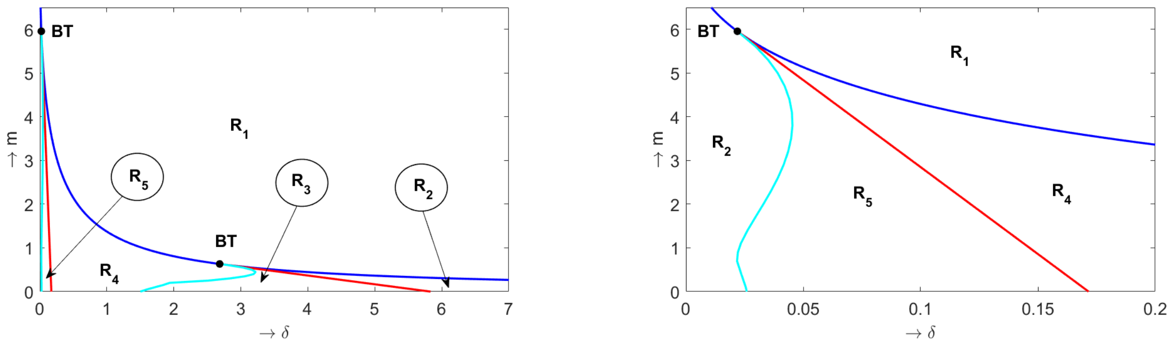

(a) (b)

Figure 4: S. (a) Two-parametric bifurcation diagram of the system (14) in δ-m parametric plane at $\alpha = 8$, (b) zoomed version (a) near origin. Description of all the curves are given in the text.

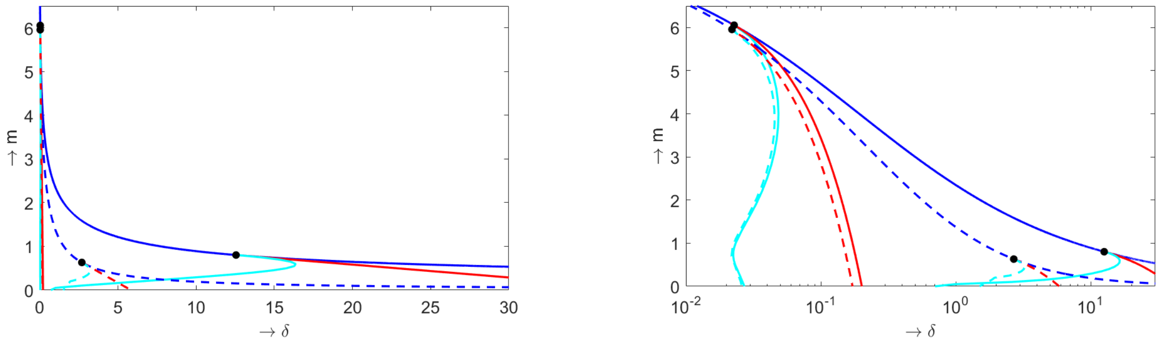

(a) (b)

Figure 5: S. (a) Combined two-parametric bifurcation diagram of the system (13) and (14) in δ-m parametric plane at fixed $\alpha = 8$, (b) same bifurcation diagram with x-axis in logarithmic scale. Solid curves corresponds to system (13) and dashed curve corresponds to system (14)

In Fig. 5S we have plotted all the local and global bifurcation curves for the two systems compositely to compare their dynamics. We use solid lines for the system (13) with Allee function in both functional and numerical responses and dashed line for the other. It is clearly seen that diagrams look like same except for the area of some regions (R_i) being increased or decreased. The way of appearance of the local and global bifurcation curves are almost same in both cases which leads the diagrams look like similar. But one noticeable fact is that predator in system (13) can persists in larger domain (in R_1) compared to the system (14) with Allee function in numerical response only. Thus the mechanism through which Allee effect appears into the predator growth, either due to exploitation of resources or lack of mating partner or something else, has a strong influence on predator persistence and can be considered as separate interesting study.
